# Detection and evaluation of signals for immune-related adverse events: a nationwide, population-based study

**DOI:** 10.3389/fonc.2023.1295923

**Published:** 2024-01-26

**Authors:** Eo Jin Kim, Ye-Jee Kim, Ja Yoon Heo, Minju Kim, Soohyeon Lee, Seyoung Seo, Jisun Myung, Ji Seon Oh, Sook Ryun Park

**Affiliations:** ^1^ Division of Hematology/Oncology, Department of Internal Medicine, Kangbuk Samsung Hospital, Sungkyunkwan University School of Medicine, Seoul, Republic of Korea; ^2^ Department of Clinical Epidemiology and Biostatistics, Asan Medical Center, University of Ulsan College of Medicine, Seoul, Republic of Korea; ^3^ Department of Internal Medicine, National Health Insurance Service Ilsan Hospital, Goyang, Republic of Korea; ^4^ Division of Oncology-Hematology, Department of Internal Medicine, Korea University College of Medicine, Korea University Anam Hospital, Seoul, Republic of Korea; ^5^ Department of Oncology, Asan Medical Center, University of Ulsan College of Medicine, Seoul, Republic of Korea; ^6^ Big Data Research Center, Asan Institute of Life Science, Asan Medical Center, Seoul, Republic of Korea; ^7^ Department of Information Medicine, Asan Medical Center, University of Ulsan College of Medicine, Seoul, Republic of Korea

**Keywords:** immune checkpoint inhibitor, immune-related adverse event, drug safety surveillance, pharmacovigilance, data mining

## Abstract

**Background:**

Immune checkpoint inhibitors (ICIs) are one of the main pillars of cancer therapy. Since other studies such as clinical trial and retrospective study have limitations for detecting the immune-related adverse events (irAEs) characterized by unpredictable onset, nonspecific symptoms and wide clinical spectrum, we aimed to identify the incidence of irAEs and to detect and evaluate the signals using real-world data.

**Methods:**

Cancer patients treated with anticancer medications were analyzed using the nationwide health insurance claims database of South Korea from 2017 to 2019, and Clinical Data Warehouse (CDW) database of Asan Medical Center (AMC), a tertiary referral hospital, from 2012 to 2019. AEs of ICI users were compared with those of non-ICI anticancer medication users. PD-1 inhibitors (nivolumab and pembrolizumab) and PD-L1 inhibitors (atezolizumab) were evaluated. We defined an AE as a newly added diagnosis after the ICI prescription using an ICD-10 diagnostic code. A signal was defined as an AE that was detected by any one of the four indices of data mining: hazard ratio (HR), proportional claims ratio (PCR), claims odds ratio (COR), or information component (IC). All detected signals were reviewed and classified into well-known or potential irAEs. Signal verification was performed for targeted AEs using CDW of AMC using diagnostic codes and text mining.

**Results:**

We identified 118 significant signals related to ICI use. We detected 31 well-known irAEs, most of which were endocrine diseases and skin diseases. We also detected 33 potential irAEs related to disorders in the nervous system, eye, circulatory system, digestive system, skin and subcutaneous tissues, and bones. Especially, portal vein thrombosis and bone disorders such as osteoporosis with pathological fracture and fracture of shoulder, upper arm, femur, and lower leg showed high HR in ICI users than in non-ICI users. The signals from hospital database were verified using diagnostic codes and text mining.

**Conclusion:**

This real-world data analysis demonstrated an efficient approach for signal detection and evaluation of ICI use. An effective real-world pharmacovigilance system of the nationwide claims database and the EMR could complement each other in detecting significant AE signals.

## 1 Introduction

Immune checkpoint inhibitors (ICIs), which activates the immune system to fight against cancer, have evolutionally changed the recent trend of cancer therapy. Monoclonal antibodies targeting T-cell immune checkpoint molecules, including programmed cell death protein 1 (PD-1), programmed cell death ligand-1 (PD-L1), and cytotoxic T lymphocyte associated protein-4 (CTLA-4), are the most effective examples of ICIs. Randomized clinical trials involving ICIs have demonstrated the durable clinical benefits of these medications across a diverse range of cancers (1–3).

However, patients treated with ICI may experience immune-related adverse events (irAEs), which are autoimmune disease-like toxicities caused by immune reaction associated with this treatment. These events exhibit distinct characteristics compared to those observed with conventional cytotoxic chemotherapy or targeted agents. They can affect any part of the body with diverse severity and onset times (4, 5). The unique profile of irAEs, characterized by unpredictable onset, even after the completion of treatment, nonspecific symptoms, a broad clinical spectrum, and in some cases, irreversibility and fatality poses a significant challenge in the pharmacovigilance of ICIs. Clinical trials have reported various adverse events (AEs), but they have limitations since they collect data from a limited number of selected populations for a restricted duration of time. In particular, ICIs have often received accelerated approval from regulatory agencies based on results from earlier phase studies with small samples sizes and short study durations (6, 7). This may increase the risk of the AEs not being fully characterized in pre-marketing clinical trials. Therefore, post-marketing pharmacovigilance of ICIs is particularly crucial to capture real-world experiences. Several efforts have been undertaken to identify AE signals using post-market pharmacovigilance databases (8–13). However, these databases primarily consist of voluntary AE reports, making them susceptible to various reporting biases and rendering them inadequate for calculating AE incidence. Large-scale observational studies using real-world data, such as electronic health records, medical claims and billing data, and registries, have the potential to overcome the inherent limitations of clinical trials and spontaneous reporting databases. Given that the National Health Insurance (NHI) system in South Korea provides healthcare coverage to the entire population residing the country and that all reimbursing claims with diagnostic codes are evaluated by the Health Insurance Review and Assessment Service (HIRA), the claim data of HIRA can be considered an appropriate data source for pharmacovigilance research. Several attempts have been made to utilize health insurance claims data as a pharmacovigilance tool for conducting post-marketing surveillance, employing multiple data mining methods (14, 15).

In the present study, our objective was to monitor known irAEs in a real-world setting and identify potential new AE signals in patients undergoing treatment with ICIs. To achieve this, we utilized a nationwide, population-based claim database as a primary data source and further validated these signals using a clinical data warehouse.

## 2 Methods

### 2.1 Data source for signal detection

We defined the potential risk associated with ICI usage as a “signal”, a term frequently used in pharmacovigilance. This term denotes “Information originating from one or several sources, indicating a possible new causal relationship or a novel aspect of a known association between an intervention and an event or a group of related events, whether adverse or beneficial” (16, 17). Signal detection in this study was conducted using the HIRA database. The HIRA database includes all information on approximately 50 million people, the whole Korean population covered by the NHI program since 2000. Every resident in South Korea is provided with a unique civil registration number. The NHI program provides coverage for all residents in a form of compulsory social insurance, which ensured the complete follow-up of study participants. In South Korea, the NHI system started to reimburse ICI treatment for non-small cell lung cancer (NSCLC) and bladder cancer from August 2017, and for melanoma from February 2018 (18). We obtained the claims data of patients with NSCLC, melanoma, and bladder cancer that had been submitted by healthcare providers between January 1, 2014, and January 31, 2019, with anonymized identifiers provided by the HIRA to protect their privacy, in accordance with the Act on the Protection of Personal Information Maintained by Public Agencies. The database contains information on demographic records, diagnosis, procedures performed, and prescriptions. Demographic information included age and sex. All diagnoses were coded using the Korean Standard Classification of Disease, seventh edition (KCD-7), a modified version of the International Classification of Disease and Related Health Problems, 10th revision (ICD-10). The prescription data included generic name of the drug according to the HIRA drug formulary code, prescription date, and duration. Access to the HIRA database was limited to researchers who completed an application process and were granted approval for research access.

### 2.2 Ethics

This study was approved by the Institutional Review Board of Asan Medical Center (IRB No. 2019-1300, 2019-1247), and informed consent was waived.

### 2.3 Study subjects for signal detection

In this study, we retrospectively identified patients with NSCLC, bladder cancer, and melanoma by using the KCD-7 codes for primary and secondary diagnoses (C34, C67, and C43, respectively), as well as the corresponding cancer-specific deductible insurance codes (V027 for NSCLC, V193 for bladder cancer, and V194 for melanoma). These patients were then categorized into two groups: the ICI group and the non-ICI group. Utilizing the 5-year HIRA claims database (from January 1, 2014, to January 31, 2019), we selected patients who received anticancer medication between August 2017 and January 2019, based on the initiation date (August 2017) of the NHI coverage for ICIs. The ICI group consisted of patients who received at least one cycle of treatment with PD-1 inhibitors (nivolumab and pembrolizumab) or PD-L1 inhibitor (atezolizumab) inhibitor between August 2017 and January 2019. The non-ICI group, serving as the comparator, included patients who received non-ICI systemic anticancer agents such as cytotoxic chemotherapy and molecular targeted therapy (**Supplementary Table S1**) for NSCLC, bladder cancer, and melanoma during the same period as the ICI group. The index date for each patient was defined as the first prescription date of the corresponding drug, and all study patients were followed up for a maximum of 18 months from the index date. AEs in the ICI group were compared with those in the non-ICI anticancer medication group.

### 2.4 Definition of adverse events

In this study, AEs were identified from the claims study database by considering newly added diagnosis codes during treatment. AEs were defined as diagnoses that did not exist prior to exposure to the drug of interest but were added after exposure. The KCD-7 coding system, which consists of 21 chapters with various code blocks, was used for classification. Physicians in South Korea are required to report the most specific diagnosis code available according to the KCD-7 coding system, ranging from three to seven digits, in the medical care cost statements submitted to the NHI system.

Initially, a low-level classification was performed using three-digit KCD codes, resulting in a group of 1,348 codes. Subsequently, a mid-level classification was conducted using groups of three-digit KCD codes to categorize single conditions within each block, resulting in a total of 222 codes. However, certain disease categories were excluded from the study. The neoplasm category (KCD-7 codes, C00-D48) was excluded due to the difficulty in distinguishing it from existing cancers, which are already indications for ICIs. Additionally, pregnancy-related diagnosis including ‘‘Pregnancy, childbirth, and the puerperium’’ (O00-O99), ‘‘Certain conditions originating in the perinatal period’’ (P00-P96), and ‘‘Congenital malformations, deformations, and chromosomal abnormalities’’ (Q00-Q99) were excluded to focus on non-pregnant cancer patients.

### 2.5 Signal detection criteria

The hazard ratio (HR) was computed by dividing the incidence of ICI-specific AE pairs (A) by the total observed duration of claims (T_1_), and then dividing the results by the incidence of non-ICI-AE pairs (C) divided by the total observed duration of claims (T_0_). If the lower limit of the 95% confidence interval (CI) of an AE’s HR was greater than 1, it was considered a potential signal possibly associated with ICIs.

Disproportionality measures were also employed to detect AE signals, using proportional claims ratios (PCRs), claims odds ratios (CORs), and Bayesian confidence propagation neural networks (BCPNNs) of information components (ICs). These measures were specifically adapted for use with the claims database, as opposed to spontaneous reporting databases. Two-by-two tables were constructed, combining the frequency of drug–AE pairs and total claims, along with the estimated indices for each AE (**Table 1**).

**Table 1:** Study framework of the disproportionality analysis of the health insurance claims database.

Drug exposure	Specific AE	Non-specific AE	Total claims time
ICI	A	B	T_1_
Non-ICI	C	D	T_0_

AE, adverse event; ICI, immune checkpoint inhibitors.

PCR is defined as the ratio between the incidence of a specific AE associated with a particular ICI and the incidence of AEs related to non-ICI drugs listed in the database (19). COR represents the odds ratio of developing a specific AE compared to all other events for the ICI group compared with the odds of developing AEs when using non-ICI drugs listed in the database (18, 19). A signal was deemed significant if the lower limit of the 95% CIs for PCR or COR was greater than 1, or if the number of ICI-specific AE pairs (A) was equal to or greater than 3. For the BCPNN analysis, a signal was considered statistically significant if the lower limit of 95% credible interval was above 0 (20) (**Table 2**).

**Table 2:** Definition and signal detection criterion for data mining indices.

Indices	Definition	Detection criterion
Hazard ratio	(A/T_1_)/(C/T_0_)	Lower limit of 95% CI>1, A≥3
Proportional claims ratio	{A/(A+B)}/{C/(C+D)}	Lower limit of 95% CI>1, A≥3
Claims odds ratio	(A/B)/(C/D)	Lower limit of 95% CI>1, A≥3
Information component	log_2_P(AE, Drug)/P(AE)P(Drug)	Lower limit of 95% credible interval≥0, A, C≥1

CI, confidence interval; P, probability.

See **Table 1** for what A, B, C, and D mean.

In this study, any AE that was detected by at least one of the four indices (HR, PCR, COR, and IC) was considered a signal. The statistical analyses were conducted using SAS Enterprise Guide (version 7.1; SAS institute, NC, USA) and R version 3.6.2 for Windows (R Foundation for Statistical Computing, Vienna, Austria). Significant signals only in the ICI group compared to the non-ICI group were presented.

### 2.6 Signal categorization

The detected AEs from the HIRA database were reviewed independently by two medical oncologists. They assessed the expectedness and frequency of the AEs compared to previous literature (21, 22). The detected AEs were categorized based on the following conditions: 1) Well-known irAEs that were established as related to immunotherapy based on previous literature, and/or are associated with the pathogenesis of the disease entity involving autoimmune activation; 2) potential irAEs that were not previously recognized as irAEs, but were considered clinically relevant and important.

### 2.7 Signal verification

Signal verification was conducted using the Clinical Data Warehouse database of Asan Medical Center (AMC) to validate targeted AEs. This validation involved utilizing diagnostic codes and text mining techniques. Recognizing the limitation of the HIRA database, which relies on health insurance claims data, the electronic medical record (EMR) from the tertiary care hospital, AMC, was employed to verify targeted AEs based on symptoms or laboratory abnormalities. The research team, consisting of clinical experts, reviewed and discussed the detected signals to choose the targeted AEs. Access to the CDW database is restricted to in-hospital researchers who have received approval from both the Institutional Review Board and the Data Subcommittee.

Among 21,205 cancer patients diagnosed with NSCLC, melanoma, or bladder cancer between July 1, 2012 and January 31, 2019 from the hospital EMR database, 9236 patients who received systemic anticancer therapy were identified using diagnostic and prescription codes (**Supplementary Figure S1**). These patients were classified into two groups: ICI users (n=836) and non-ICI users (n=8,400). The observation period for all participants commenced from their respective first prescription date, and they were followed up until the end of the study period.

In addition, we identified the other validation group of 1,706 ICI users from the hospital EMR database, comprising patients diagnosed with any type of cancer, for clinical text analysis (text mining). This validation group included patients who were not reimbursed for ICI treatment. Then, these 1,706 ICI users were matched in a 1:2 ratio to 3,412 non-ICI users based on age, sex, and cancer subtype to compare the detailed information documented in the narrative chart.

Among the detected AE signals, targeted AEs that could be easily detected using diagnostic codes or diagnostic codes required on prescriptions were searched using the diagnostic code information (‘diagnostic code from the hospital EMR data’, n=9236). On the other hand, text mining techniques were applied to search for targeted AE signals that could be easily detected in narrative EMR records such as nursing records and progression notes (‘text mining applied to the hospital EMR data’, n=5118; 1,706 ICI users and 3,412 non-ICI users). Thus, there were not only signals that were verified by both methods, but also other signals excluded from each analysis.

To explore the unstructured data obtained from the EMR, including the clinical notes written by medical staff, we utilized Standard Query Language with the LIKE function in the R package (SQLDF) to identify meaningful words. The operational definition of the verified signals, including the disease category and detailed search terms, can be found in **Supplementary Table S2**.

## 3 Results

### 3.1 Characteristics of the study subjects

A total of 314,423 patients were diagnosed with NSCLC, melanoma, or bladder cancer between July 2014 and January 2019. Among these patients, 25,817 were prescribed with anticancer medications from August 2017 to January 2019. Among them, 4,516 patients received ICIs, while 21,301 patients received non-ICI anticancer medications (**Supplementary Figure S2**).

Baseline characteristics of the study patients are shown in **Supplementary Table S3**. While there were statistically significant differences between the ICI group and the non-ICI group in all variables, the differences were not clinically significant. Overall, 74.1% of the patients were male, with a mean (± standard deviation; SD) age of 66.4 (± 10.0) years and a mean (± SD) Charlson comorbidity index (CCI) of 7.5 (± 3.6). The majority of patients had NSCLC (88.5%), while bladder cancer and melanoma accounted for 9.0% and 2.6% of the cases, respectively. In the ICI group, 75.6% of the patients were male, with a mean (± SD) age of 64.9 (± 10.0) years and a mean (± SD) CCI of 8.6 (± 3.6). The majority of patients in this group had NSCLC (83.1%), while bladder cancer and melanoma accounted for 11.4% and 5.6% of the cases, respectively.

### 3.2 Number of detected signals from the National Health Insurance claims database

**Figure 1:**
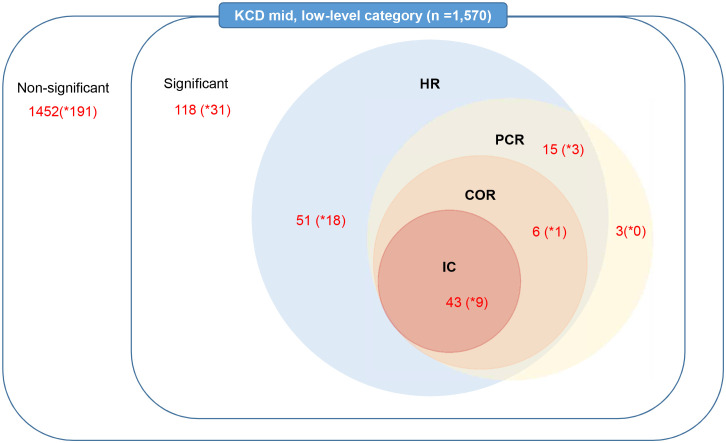
Number of detected signals related to immune checkpoint inhibitor use by data-mining indices in the nationwide claims database. HR, hazard ratio; PCR, proportional claims ratio; COR, claims odds ratio; IC, information component; KCD, Korean Standard Classification of Diseases. * means number of detected signals in mid-level.

**Figure 1** illustrated the number of detected signals and the logical relations in which the detection criterion was satisfied by each index. Out of the 1,570 screened AEs using the KCD code, a total of 118 distinct AEs were identified as signals associated with ICIs using the HR index (n=115), PCR index (n=67), COR index (n=49), and IC index (n=43). The HR index demonstrated the highest number of detections, followed by PCR, COR, and IC, in descending order.

### 3.3 Detected AE signals

#### 3.3.1 Incidence of detected AE signals

When detected AEs were categorized by the organ system, the ICI group exhibited the highest incidence of AEs in the endocrine, respiratory, dermatologic, and musculoskeletal systems, which included disorders in the thyroid gland (E00–E07, 20.8%), other disorders of the thyroid gland (E07, 16.0%), influenza and pneumonia (J09–J18, 21.1%), pneumonia, (J18, 18.6%), pruritus, (L29, 11.4%), and arthropathies (M00–25, 17.5%) (**Table 3**; **Supplementary Table S4**). Mental and behavioral disorders (F00–99) as well as infectious and parasitic diseases (A00–B39) were also common AEs observed in the ICI group, ranging from 5% to 10% in incidence. Hepatic organ-related AEs (K71) were present in approximately 4% of the ICI group. Other AEs, such as neurologic (G00–99), circulatory (I00–99), and digestive diseases (K00–93), were relatively infrequent, with an incidence of less than <5%. The **Table 3** displays 43 AE signals that were found to be significant in all four criteria (HR, PCR, COR, IC) among the 118 detected signals of AEs associated with the use of ICIs in the HIRA database. These signals met the threshold for significance across all four indices, indicating a strong association with ICI treatment.

**Table 3:** Adverse event signals significant in all four criteria (HR, PCR, COR, IC) in the HIRA database.

	KCD code	English	ICI user		Non-ICI-user		HR			PCR			COR			IC**
			AE (%)	Total	AE (%)	Total	point estimates	lower CI	upper CI	point estimates	lower CI	upper CI	point estimates	lower CI	upper CI	
IV. Endocrine, nutritional and metabolic diseases (E00-E90)																
W*	E00–E07	Disorders of the thyroid gland	20.8	2,784	7.6	14,323	3.96	3.58	4.38	2.73	2.52	2.94	3.18	2.85	3.55	0.94
W	E03	Other types of hypothyroidism	5.9	4,068	1.7	19,313	4.38	3.71	5.17	3.4	3.08	3.76	3.55	3	4.21	1.04
W	E05	Thyrotoxicosis [hyperthyroidism]	1.4	4,344	0.5	20,454	3.1	2.26	4.25	2.57	2.09	3.15	2.59	1.89	3.55	0.59
W	E06	Thyroiditis	1	4,409	0.4	20,742	3.34	2.31	4.82	2.71	2.15	3.43	2.73	1.89	3.95	0.58
W	E07	Other disorders of thyroid	16	3,280	5.8	16,884	3.8	3.42	4.23	2.77	2.56	2.99	3.11	2.77	3.48	0.95
	E20–E35	Disorders of other endocrine glands	3.7	4,224	2.6	20,126	1.69	1.41	2.02	1.39	1.21	1.6	1.41	1.17	1.69	0.13
P*	E24	Cushing’s syndrome	0.1	4,509	0	21,262	5.74	1.66	19.85	4.72	2.54	8.77	4.72	1.37	16.31	0.18
	E27	Other disorders of the adrenal gland	2.2	4,363	1.5	20,945	1.79	1.42	2.24	1.49	1.25	1.77	1.5	1.19	1.89	0.14
	E35	Disorders of the endocrine glands in diseases classified elsewhere	0.5	4,501	0.1	21,196	5.54	3.16	9.71	4.52	3.39	6.02	4.54	2.59	7.96	0.81
P	E83	Disorders of mineral metabolism	4.5	4,191	3.4	20,206	1.58	1.34	1.86	1.33	1.17	1.51	1.34	1.14	1.58	0.1
V. Mental and behavioral disorders (F00-F99)																
P	F05	Delirium, not induced by alcohol and other psychoactive substances	3.9	4,409	3.2	21,017	1.48	1.25	1.75	1.23	1.08	1.41	1.24	1.05	1.47	0.01
VI. Diseases of the nervous system (G00-G99)																
P	G40	Epilepsy	3.8	4,107	3	20,088	1.58	1.33	1.88	1.29	1.12	1.49	1.3	1.09	1.56	0.05
	G80–G83	Cerebral palsy and other paralytic syndromes	2.2	4,362	1.5	20,811	1.77	1.4	2.22	1.46	1.22	1.74	1.47	1.17	1.85	0.12
	G81	Hemiplegia	1.6	4,420	1	20,971	1.94	1.47	2.55	1.6	1.31	1.97	1.61	1.23	2.13	0.16
	G83	Other paralytic syndromes	0.3	4,482	0.1	21,187	2.65	1.34	5.26	2.18	1.37	3.49	2.18	1.1	4.33	0.01
	G93	Other disorders of the brain	0.8	4,449	0.5	21,015	1.93	1.32	2.83	1.59	1.19	2.12	1.59	1.09	2.34	0.02
VII. Diseases of the eye and adnexa (H00-H59)																
P	H28	Cataract and other disorders of lens in diseases classified elsewhere	0.1	4,514	0	21,256	5.82	1.45	23.27	4.71	2.35	9.42	4.71	1.18	18.85	0.06
**IX. Diseases of the circulatory system (I00-I99)**																
P	I46	Cardiac arrest	4.4	4,490	2.7	21,243	1.97	1.67	2.31	1.64	1.45	1.85	1.67	1.41	1.97	0.33
P	I81	Portal vein thrombosis	0.1	4,515	0	21,291	5.57	1.79	17.28	4.72	2.68	8.31	4.72	1.52	14.64	0.27
X. Diseases of the respiratory system (J00-J99)																
	J90–J94	Other diseases of pleura	9.3	3,501	7.1	17,681	1.53	1.36	1.73	1.3	1.17	1.43	1.33	1.17	1.51	0.13
W	J90	Pleural effusion, NEC	7.2	3,706	5.5	18,540	1.55	1.35	1.77	1.3	1.17	1.46	1.33	1.15	1.53	0.12
	J91	Pleural effusion in conditions classified elsewhere	1.9	4,369	1.3	20,865	1.72	1.34	2.21	1.45	1.19	1.76	1.46	1.13	1.87	0.08
XI. Diseases of the digestive system (K00-K93)																
	K87	Disorders of gallbladder, biliary tract, and pancreas in diseases classified elsewhere	0.4	4,494	0.1	21,253	5.34	2.75	10.37	4.47	3.17	6.29	4.48	2.31	8.70	0.68
XII Diseases of the skin and subcutaneous tissue (L00-L99)																
W	L43	Lichen planus	0.1	4,501	0	21,245	11.48	2.87	45.95	9.44	5.94	14.99	9.45	2.36	37.81	0.58
W	L51	Erythema multiforme	0.2	4,497	0	21,249	5.57	2.32	13.39	4.73	3.05	7.33	4.73	1.97	11.38	0.51
P	L74	Eccrine sweat disorders	0.1	4,506	0	21,239	5.7	1.65	19.71	4.71	2.53	8.77	4.72	1.37	16.30	0.17
	L80–L99	Other disorders of the skin and subcutaneous tissue	11	3,633	8.3	18,038	1.58	1.42	1.77	1.33	1.21	1.46	1.37	1.22	1.54	0.17
W	L80	Vitiligo	0.3	4,484	0	21,212	17.66	5.69	54.79	14.19	10.68	18.86	14.23	4.59	44.13	1.07
P	L89	Decubitus ulcer and pressure area	8.8	4,305	6.2	20,806	1.68	1.5	1.88	1.41	1.29	1.55	1.45	1.29	1.63	0.23
XIII. Diseases of the musculoskeletal system and connective tissue (M00-M99)																
	M30–M36	Systemic connective tissue disorders	0.5	4,439	0.3	20,965	2.39	1.47	3.89	1.94	1.37	2.74	1.94	1.2	3.16	0.11
W	M32	Systemic lupus erythematosus	0.1	4,502	0	21,219	4.92	1.65	14.65	4.04	2.24	7.27	4.04	1.36	12.04	0.18
W	M35	Other systemic involvement of connective tissue	0.4	4,463	0.2	21,090	2.77	1.53	5.01	2.22	1.48	3.33	2.23	1.23	4.04	0.12
P	M84	Disorders of continuity of bone	0.9	4,454	0.5	21,148	2.16	1.49	3.13	1.77	1.35	2.32	1.78	1.22	2.58	0.15
XVIII. Symptoms, signs and abnormal clinical and laboratory findings, not elsewhere classified (R00-R99)																
	R18	Ascites	1.4	4,454	0.9	21,095	1.88	1.41	2.51	1.56	1.25	1.94	1.57	1.17	2.10	0.11
	R44	Other symptoms and signs involving general sensations and perceptions	0.1	4,502	0	21,264	5.89	1.88	18.42	4.72	2.68	8.32	4.73	1.52	14.67	0.27
	R95–R99	Ill-defined and unknown causes of mortality	0.6	4,514	0.3	21,297	2.4	1.53	3.77	1.99	1.45	2.73	2	1.27	3.13	0.18
	R99	Other ill-defined and unspecified causes of mortality	0.5	4,514	0.3	21,297	2.48	1.53	4.01	2.06	1.47	2.88	2.06	1.28	3.34	0.17
XIX. Injury, poisoning and certain other consequences of external causes (S00-T98)																
P	S32	Fracture of the lumbar spine and pelvis	1.6	4,343	1	20,523	1.97	1.51	2.58	1.59	1.3	1.95	1.6	1.22	2.10	0.16
P	S42	Fracture of the shoulder and upper arm	0.4	4,463	0.2	21,124	2.62	1.48	4.65	2.12	1.43	3.15	2.12	1.2	3.76	0.1
	S70–S79	Injuries to the hip and thigh	1.7	4,152	1.2	19,599	1.8	1.38	2.35	1.48	1.2	1.81	1.48	1.13	1.94	0.08
	S70	Superficial injury of the hip and thigh	0.7	4,393	0.3	20,702	2.34	1.52	3.61	1.92	1.41	2.62	1.93	1.25	2.98	0.16
P	S72	Fracture of the femur	0.7	4,460	0.4	21,075	2.21	1.45	3.36	1.82	1.34	2.47	1.82	1.2	2.78	0.11
XX. External causes of morbidity and mortality (V01-Y98)																
	X50–X57	Overexertion, travel, and privation	0.1	4,515	0	21,295	16.98	1.76	163.40	14.15	8.03	24.93	14.16	1.47	136.15	0.26

*W is for Well-known immune-related AEs (dark shade), and P is for Potential ICI-related AEs (light shade).

**The IC value is statistically significant when it is greater than zero.

KCD, Korean Standard Classification of Diseases; AE, adverse events; HR, hazard ratio; CI, confidence interval; PCR, proportional claims ratio; COR, claims odds ratio; IC, information component lower credible interval, NEC; not elsewhere classified.

#### 3.3.2 Well-known immune-related AEs

**Figure 2:**
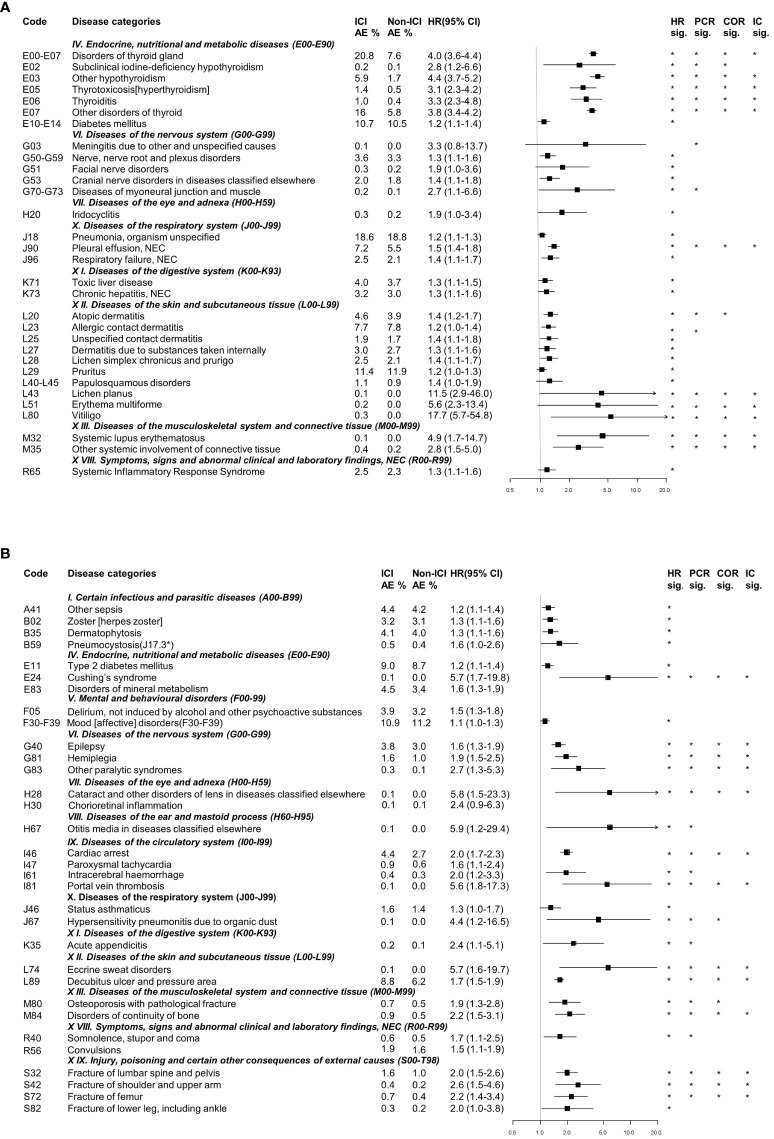
Categorization of detected signals related to immune checkpoint inhibitor use from the nationwide claims database. **(A)** Well-known immune-related adverse events. **(B)** Potential immune-related adverse events. AE, adverse events; HR, Hazard ratio; CI, Confidence interval; PCR, Proportional Claims Ratio; COR, Claims Odds Ratio; IC, Information Component. * detected statistically significant results.

Well-known irAEs were identified in endocrine and skin diseases in association with the use of ICIs (**Figure 2A**). The incidence of endocrine diseases was significantly higher in patients receiving ICIs, including endocrine gland disorders (HR: 5.5, 95% CI: 3.2–9.7), other types of hypothyroidism (HR: 4.4, 95% CI: 3.7–5.2), disorders of the thyroid gland (HR: 4.0, 95% CI: 3.6–4.4), and other disorders of the thyroid (HR: 3.8, 95% CI: 3.4–4.2). Furthermore, the use of ICIs was strongly associated with skin disease, such as vitiligo (HR: 17.7, 95% CI: 5.7–54.8), lichen planus (HR: 11.5, 95% CI: 2.9–46.0), and erythema multiforme (HR: 5.6, 95% CI: 2.3–13.4). In addition, as is well known, connected tissue disorders, including systemic lupus erythematosus (HR: 4.9, 95% CI: 1.7-4.9) and other systemic involvement of connective tissue (HR: 2.8, 95% CI: 1.5-2.8), as well as and nervous disease such as nerve, nerve root, and plexus disorders (HR: 1.3, 95% CI: 1.1–1.6), were detected with a significant association with ICI use.

#### 3.3.3 Potential ICI-related AE

Detected AEs that were not previously well-known but judged as clinically relevant and important were defined as potential ICI-related AEs. The following associations were identified: i) Infection-related AEs-other types of sepsis, herpes zoster, dermatophytosis, and pneumocystosis; ii) Endocrine-related AEs- type 2 diabetes mellitus, Cushing’s syndrome and disorders of mineral metabolism; iii) Mental and behavioral disorders-related AEs-delirium and mood disorders; iv) Eye and adnexa-related AEs-cataract, chorioretinal inflammation, and otitis media; v) Circulatory system-related AEs-cardiac arrest, intracerebral hemorrhage, and portal vein thrombosis; vi) Respiratory system-related AEs-status asthmaticus and hypersensitivity pneumonitis due to organic dust; vii) Digestive system-related AEs-acute appendicitis; viii) Skin and subcutaneous tissue-related AEs-eccrine sweat disorders and decubitus ulcer; and viii) Bone disorders-related AEs-osteoporosis with pathological fracture, disorders in the continuity of the bone, fracture of the shoulder and upper arm, fracture of the femur, and fracture of the lower leg, including the ankle (**Figure 2B**).

### 3.4 Signal verification with hospital EMR database

**Figure 3:**
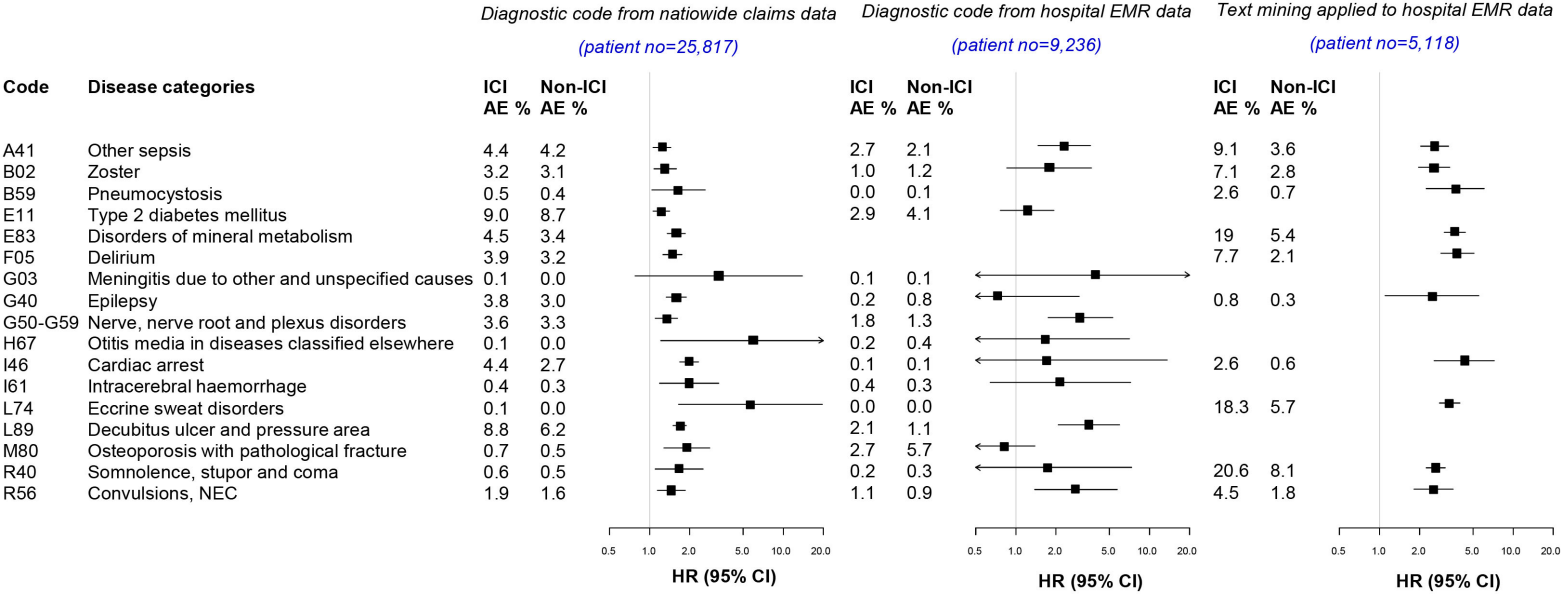
Signal verification from the nationwide claims database and hospital electronic medical record database. AE, adverse events; HR, Hazard ratio; CI, Confidence interval,.

Among the detected AE signals, signal verification for 17 targeted AEs was performed using additional hospital EMR database to address the limitation of the HIRA claims data (**Figure 3**; **Supplementary Table S2**). The incidence of detected AE signals was compared between population-level and hospital-level database. The signal verification was performed in two ways, using the diagnostic code and text mining.

From diagnostic codes in hospital-level EMR data (n=9,236; 836 ICI users and 8,400 non-ICI users), other types of sepsis (HR 2.3, 95% CI 1.5–3.6), nerve root and plexus disorders (HR 3.0, 95% CI 1.7–5.3), decubitus ulcer (HR 3.5, 95% CI 2.1–6.0), and convulsion (HR 2.8, 95% CI 1.4–5.7) were identified (**Figure 3**).

After defining targeted AEs by search term through text mining aside from using diagnostic codes (**Supplementary Table S2**), AE signals were verified from the hospital EMR database (**Figure 3**). In patients available for text mining (n=5118; 1,706 ICI users and 3,412 non-ICI users), the detected AEs were i) related to infections: other types of sepsis, herpes zoster, and pneumocystis; ii) related to metabolic disease: disorders in mineral metabolism; iii) related to mental disorders: delirium; iv) related to nervous disease: epilepsy; v) related to circulatory system: cardiac arrest: vi) related to skin and subcutaneous tissue: eccrine sweat disorders; and vii) related to symptom: somnolence and convulsion. These results indicate that AE signals detected from the NHI database were verified from the hospital EMR database, using both diagnostic codes and text mining.

## 4 Discussion

In the present study, we utilized both nationwide claims databases and hospital EMR to comprehensively monitor and detect signals of irAEs possibly associated with ICIs. The hospital EMR data, which includes structured and unstructured data such as clinical notes, was utilized to verify the detected irAE signals in the claim data. By integrating these two different real-world databases, we were able to identify irAE signals that were not well-established in the literature and assess their association with the use of ICIs. This integrated approach highlights the importance of utilizing both nationwide claims databases and hospital EMR data in real-world pharmacovigilance system. This comprehensive approach provides valuable insights into the safety profile of ICIs and contributes to the ongoing monitoring and management of irAEs in clinical practice.

Our analysis of claim data revealed the spectrum and incidences of well-known irAEs at the population level. The most common well-known irAEs observed were thyroid disorders, such as hypothyroidism, subclinical hypothyroidism, hyperthyroidism, and thyroiditis (20.8% in the ICI group vs. 7.6% in the non-ICI group; HR = 4.0, 95% CI 3.6–4.4). The incidence of thyroid irAEs in our study appeared to be higher than reported in phase III clinical trials, where the mean incidence was around 10.8% (23), but consistent with previous real-world studies, which have reported incidence rates ranging from 13% to 30% (24–26). In addition, our claim data analysis revealed other well-known irAEs across various organ systems, such as dermatologic AEs such as pruritus, dermatitis, and atopic dermatitis, musculoskeletal AEs such as systemic lupus erythematosus and other systemic involvement of connective tissue, gastrointestinal AEs such as hepatitis, respiratory AEs such as pneumonitis, ocular AEs such as iridocyclitis, and neurologic AEs such as meningitis (**Figure 2**). The incidence of these well-known typical irAEs was similar to previous studies involving PD-1/PD-L1 inhibitors (5, 27–30). Of note, the incidence of irAEs in respiratory system we detected was higher than that of previously reported (5, 31). These findings demonstrate the utility of nationwide claims databases in real-world AE monitoring and risk measurement.

Furthermore, our analysis unveiled new AE signals occurring more frequently in patients receiving ICIs compared to those receiving non-ICI agents. In the endocrine system, we identified type 2 diabetes mellitus (DM) (9.0% in the ICI group vs. 8.7% in the non-ICI group; HR = 1.2, 95% CI 1.1–1.4) and Cushing’s syndrome (0.1% in the ICI group vs. 0.0% in the non-ICI group; HR = 5.7, 95% CI 1.7–19.8) as a new signal associated with ICI use. While ICIs are known to rarely cause type 1 DM (32, 33), their association with new-onset type 2 DM has not been well established. However, recent studies have indicated that ICIs may exacerbate pre-existing type 2 DM (33, 34) and increase the risk of developing incident diabetes (35), which aligns with our findings. Regarding Cushing’s syndrome, it has been described in case reports of patients receiving ICIs (36, 37), but the specific details and relationship between Cushing’s syndrome and ICIs have not been elucidated due to its rarity. These findings underscore the value of utilizing large-scale population-based claim data to uncover rare AE profiles. Nevertheless, caution is warranted in interpretation, as there is potential for increased steroid administration in ICI-treated patients due to irAEs. This may partially contribute to bias, leading to a higher prevalence of diabetes or Cushing ‘s disease in the ICI group compared to the non-ICI group.

In the nervous system, we detected an association between ICIs and epilepsy (3.8% in the ICI group vs. 3.0% in the non-ICI group; HR = 1.6, 95% CI 1.3–1.9), as well as somnolence/stupor/coma (0.6% in the ICI group vs. 0.5% in the non-ICI group; HR = 1.7, 95% CI 1.1–2.5). These findings were verified using hospital EMR data through text mining (for epilepsy, 0.8% in the ICI group vs. 0.3% in the non-ICI group, HR = 2.5, [95% CI: 1.8-3.5]; for somnolence/stupor/coma, 20.6% vs. 8.1%, HR = 2.6 [95% CI: 2.2-3.1)]. While these AE signals might be secondary to other well-known neurologic irAE such as encephalitis, they may also be associated with unrevealed irAE. In the circulatory system, we found new signals of cardiac arrest (4.4% in the ICI group vs. 2.7% in the non-ICI group; HR = 2.0, 95% CI 1.7–2.3), intracerebral hemorrhage (0.4% in the ICI group vs. 0.3% in the non-ICI group; HR = 2.0, 95% CI 1.2–3.3), and portal vein thrombosis (0.1% in the ICI group vs. 0.0% in the non-ICI group; HR: 5.6, 95% CI: 1.8–17.3) associated with ICI use. Thrombosis have been increasingly reported as an AE associated with ICIs, although data on its association with ICIs compared to traditional chemotherapy are conflicting (38–40). Mechanisms involving activated T cells, proinflammatory cytokines, and endothelial and platelet activation have been proposed (41–46).

Another notable finding was the association between ICIs and bone disorders, including osteoporosis with pathological fracture and fractures of various sites. After first case series describing skeletal irAEs associated with ICI in 2018 (46), another large-scale pharmacovigilance analysis using the Food and Drug Association (FDA) Adverse Event Reporting System recently suggested a possible cause-effect relationship between serious vertebral fractures and ICI use in patients without apparent preexisting risk factors (47). Emerging evidence suggests that systemic activation of T cells leads to an osteoprotegerin ligand-mediated increase in osteoclastogenesis and bone loss. ICIs can enhance bone resorption by activating T cells (48), causing bone fragility and increasing the risk of fractures (49–51).

The verification of AE signals using the hospital EMR database demonstrated the complementarity of diagnostic codes and text mining. Text mining analysis has the potential of capturing AEs that clinicians have not yet assigned a diagnosis code to, or for which diagnostic codes could not assigned. The integration of these two approaches can enhance the detection of significant AE signals.

To the best of our knowledge, this population-based study is the first to investigate the nationwide incidence of AEs and compare the incidence of AEs between users of ICIs and conventional chemotherapy. Previous large-scale studies utilizing the FDA Adverse Event Reporting System or U.S. commercial insurance database have only reported the incidence of irAEs among ICI users in observational settings (48, 52). Moreover, our study included a large number of cancer patients treated with ICIs, surpassing the sample size of most randomized controlled trials (53). In addition, we applied multiple data mining indices and conducted a comprehensive review of the identified signals. If an AE signal was detected by multiple data mining indices, it was considered more robust compared to signals detected by a single index. The majority of signals reported in our study were detected using the HR, which reflects the time until the occurrence of an event. Consistent with our findings, another study that utilized a national health insurance claims database reported that the HR exhibited the highest sensitivity among the data mining indices (15). Therefore, it is recommended to apply multiple indices and compare the results, as it is challenging to determine the superior method of data mining (15, 54). Finally, we validated the signals identified from the hospital EMR database using two approaches: diagnostic codes and text mining.

Our study has several limitations. Firstly, due to the observation nature of this study, there is a possibility of misclassifying AEs. Since AEs were defined based on diagnostic codes, symptoms or non-diagnosed conditions may have gone undetected. Similarly, even if keywords were found in the hospital records, we cannot be certain if AEs actually occurred. Secondly, we were unable to assess the severity of AEs and detailed patient information such as cancer stage, performance status, and treatment history. Thirdly, it is conceivable that AEs associated with other anticancer medications may have been identified after transition to different anticancer medications within 18 months of initiating either ICI or non-ICI. Despite the potential for this bias, our intention was to capture signals for a sufficient duration after beginning study drug administration. The decision was motivated by the fact that various and significant irAEs such as endocrine AEs, colitis, nephritis or hepatitis can manifest at any time, even after the discontinuation of ICI therapy, and may wax and wane over time (55, 56). Fourthly, we only included PD1/PD-L1 inhibitors such as nivolumab, pembrolizumab, and atezolizumab. Hence, the safety profiles of other types of ICIs, such as CTLA-4 inhibitors, should be evaluated in the future. Finally, beyond signal detection and validation, which involved comparing the frequency of AEs between the ICI and non-ICI groups, we recognize the absence of in-depth analysis in this study. We are planning further research with more comprehensive investigations, including retrospective analyses using large-scale, long-term hospital databases, and analyses in prospective trials. These will aim to explore potential irAEs identified in our study, such as portal vein thrombosis and bone disorders.

## 5 Conclusion

This real-world data analysis utilizing both nationwide claims databases and hospital EMR, has demonstrated an effective approach for signal detection and evaluation of the irAEs. The development of an effective real-world pharmacovigilance system utilizing nationwide claims database can complement existing EMR systems and contribute to the early diagnosis and prompt treatment of rare irAEs. Further research and development of near real-time or earlier access to pharmacovigilance systems are warranted to enhance patient safety and improve outcomes.

## Data availability statement

The data analyzed in this study is subject to the following licenses/restrictions: To utilize the Health Insurance Review and Assessment Service (HIRA) database, a formal application and approval process on the official website 'https://opendata.hira.or.kr/home.do.' was needed. Subsequent to the data export procedure, access to the dataset was granted on a limited and temporary basis, contingent upon a thorough review by the data-holding institution, with external network access restricted. For any inquiries or requests pertaining to dataset access, please direct them to 'https://opendata.hira.or.kr/home.do.

## Ethics statement

The studies involving humans were approved by Institutional Review Board of Asan Medical Center (IRB No. 2019-1300, 2019-1247). The studies were conducted in accordance with the local legislation and institutional requirements. Written informed consent for participation was not required from the participants or the participants’ legal guardians/next of kin in accordance with the national legislation and institutional requirements.

## Author contributions

EK: Supervision, Writing – original draft, Writing – review & editing. Y-JK: Formal analysis, Methodology, Supervision, Visualization, Writing – original draft, Writing – review & editing. JH: Conceptualization, Supervision, Writing – original draft. MK: Formal analysis, Writing – original draft. SL: Conceptualization, Methodology, Writing – review & editing. SS: Conceptualization, Supervision, Writing – review & editing. JM: Data curation, Formal analysis, Writing – original draft. JO: Conceptualization, Funding acquisition, Methodology, Supervision, Writing – review & editing. SP: Conceptualization, Funding acquisition, Methodology, Project administration, Supervision, Writing – original draft, Writing – review & editing.
