# Supplementary material for: Detection and evaluation of signals for immune-related adverse events: a nationwide, population-based study
Source: Front Oncol. 2024 Jan 26;13:1295923. doi: 10.3389/fonc.2023.1295923 (PMC10854742; doi:10.3389/fonc.2023.1295923)
Supplement: Supplementary file 1 [file DataSheet_1.docx]

Supplementary Material

# Supplementary Figures and Tables

## Supplementary Figures


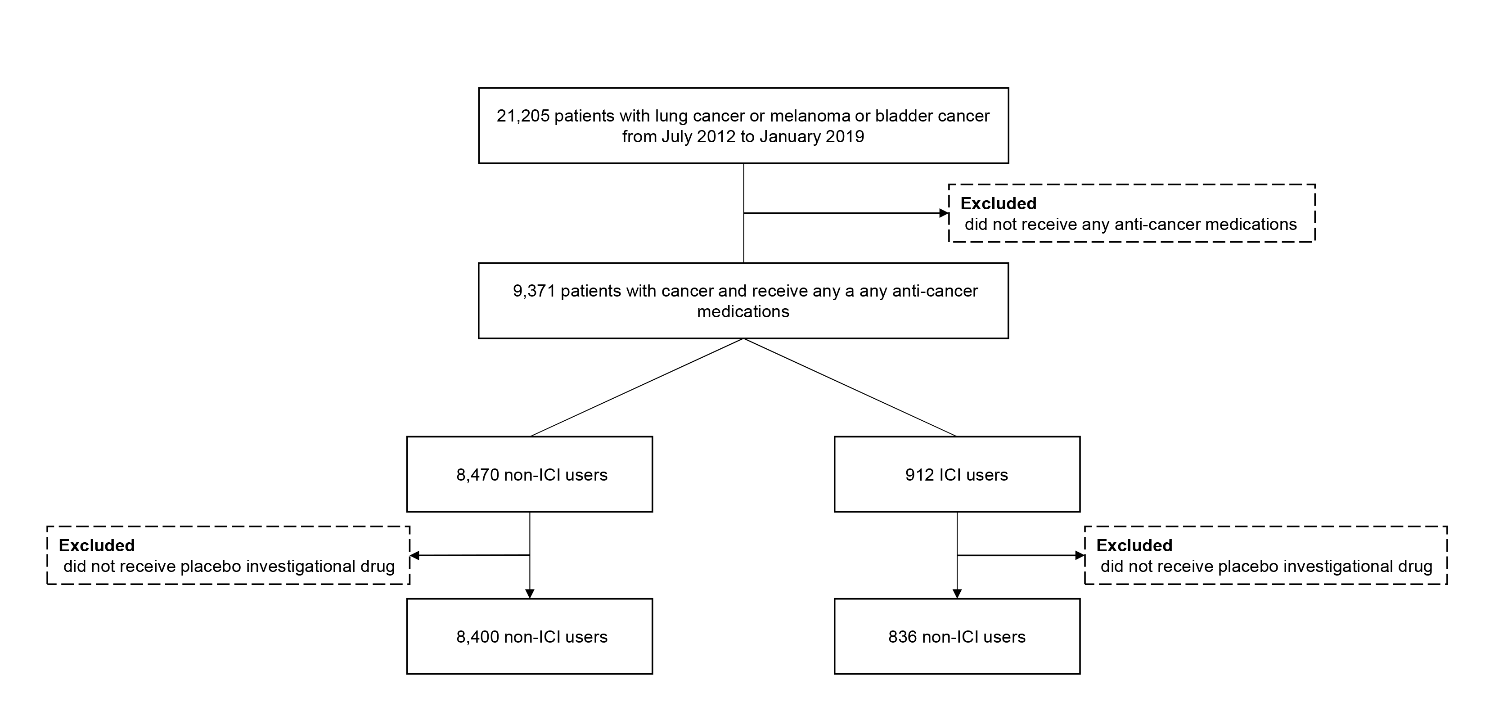
**Supplementary Figure S1.** Selection of study subject in immune checkpoint inhibitors (ICI) use and concurrent non-ICI use


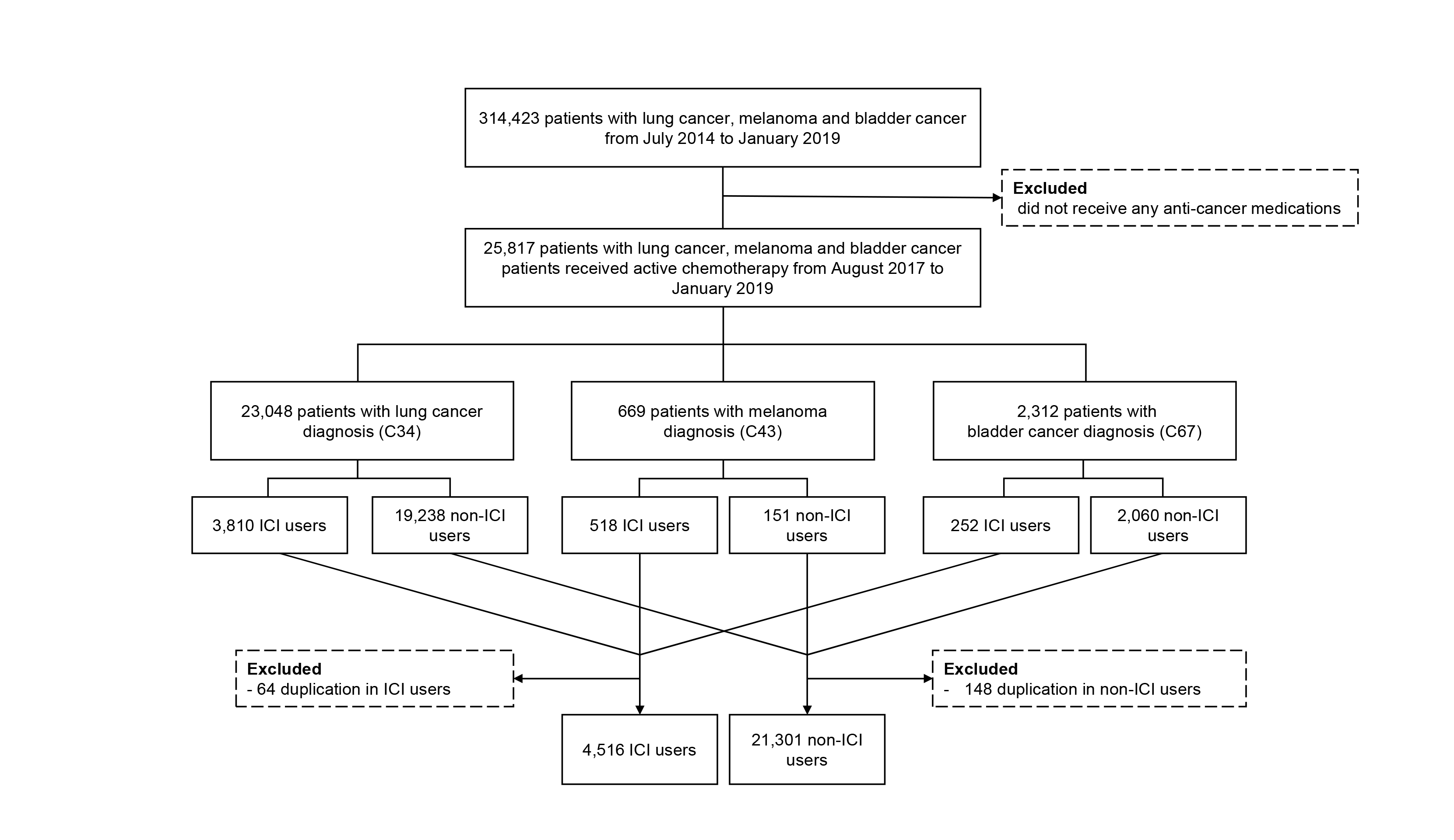


**Supplementary Figure S2.** Selection of study participants using immune checkpoint inhibitors (ICIs) and non-ICIs in the nationwide claims database

**Supplementary table S1. List of anti-cancer medication covered by national health insurance benefit program**

| **Anti-cancer medications** | | | **Cancer subtype** | | |
| --- | --- | --- | --- | --- | --- |
| **ATC code** | **ATC name** | **HIRA formulary code 4 digit** | **Lung cancer** | **Melanoma** | **Bladder cancer** |
| L01AA01 | Cyclophosphamide | 1390 | Y |  | Y |
| L01AA06 | Ifosfamide | 1733 | Y |  | Y |
| L01AX04 | Dacarbazine | 1399 |  | Y |  |
| L01BA01 | Methotrexate | 1921 |  |  | Y |
| L01BA04 | Pemetrexed | 4812 | Y |  |  |
| L01BC05 | Gemcitabine | 1649 |  |  | Y |
| L01CA01 | Vinblastine | 2478 | Y | Y | Y |
| L01CA02 | Vincristine | 2480 | Y |  |  |
| L01CA04 | Vinorelbine | 2482 | Y |  |  |
| L01CB01 | Etoposide | 1571 | Y |  |  |
| L01CD01 | Paclitaxel | 2078, 5037 | Y |  |  |
| L01CD02 | Docetaxel | 1483 | Y |  |  |
| L01DB01 | Doxorubicin | 1494 | Y |  | Y |
| L01DC01 | Bleomycin | 1180 |  | Y |  |
| L01XA01 | Cisplatin | 1345 | Y | Y | Y |
| L01XA02 | Carboplatin | 1237 | Y |  | Y |
| L01XC07 | Bevacizumab | 5543 | Y |  |  |
| L01XE02 | Gefitinib | 4530 | Y |  |  |
| L01XE03 | Erlotinib | 4774 | Y | Y |  |
| L01XE15 | Vemurafenib | 6205 |  |  |  |
| L01XE16 | Crizotinib | 6715 | Y |  |  |
| L01XE23 | Dabrafenib | 6631 |  | Y |  |
| L01XE25 | Trametinib | 6454 |  | Y |  |
| L01XE28 | Ceritinib | 6344 | Y |  |  |
| L01XE35 | Osimertinib | 6525 | Y |  |  |
| L01XE36 | Alectinib | 6562 | Y |  |  |
| L01XX17 | Topotecan | 2419 | Y |  |  |
| L01XX19 | Irinotecan | 1774 | Y |  |  |
| L01XX68 | Belotecan | 4528 | Y |  |  |
| L02BA01 | Tamoxifen | 2345 |  | Y |  |
| L03AB04 | Interferon alfa-2a | 1755 |  | Y |  |
| L01XC17 | Nivolumab | 6384 | Y | Y | Y |
| L01XC18 | Pembrolizumab | 6390 | Y | Y | Y |
| L01XC32 | Atezolizumab | 6577 | Y | Y | Y |

Abbreviations: ATC: The Anatomical Therapeutic Chemical Classification System, HIRA: Health Insurance Review and Assessment Service, Y: yes

**Supplementary Table S2. Operational definition of the verified signals related to immune checkpoint inhibitors.**

When the EMR database search for signal verification was performed with a diagnostic code, the corresponding diagnostic code was described, and when text mining with search term was done, the corresponding search term was described. There were not only signals which were verified by both methods, but also those excluded from each analysis.

| **HIRA database** | | **Signal verification with EMR database** | |
| --- | --- | --- | --- |
| **KCD code** | **Diagnosis** | ***Diagnostic code*** | ***Search term* used for Text mining**  **(full term, abbreviation, or Korean word)** |
| A41 | Other sepsis | A41 | sepsis, septic shock, Korean word of "sepsis" |
| B02 | Zoster [herpes zoster] | B02 | zoster, herpes, Korean word of "herpes zoster" |
| B59 | Pneumocystosis(J17.3*) | B59 | pneumocystis, jirovecii, carinii, PCP, PJP |
| E11 | Type 2 diabetes mellitus | E11 |  |
| E83 | Disorders of mineral metabolism |  | hypercalcemia, hypocalcemia, hyperkalemia, hypokalemia, hypernatremia, hyponatremia |
| F05 | Delirium, not induced by alcohol and other psychoactive substances |  | delirium, delirious, Korean word of delirium |
| G03 | Meningitis due to other and unspecified causes | G03 |  |
| G40 | Epilepsy | G40 | epilepsy |
| G50-G59 | Nerve, nerve root and plexus disorders | G50-G59 |  |
| H67 | Otitis media in diseases classified elsewhere | H67 |  |
| I46 | Cardiac arrest | I46 | arrest |
| I61 | Intracerebral haemorrhage | I61 |  |
| L74 | Eccrine sweat disorders | L74 | sweat, Korean word of "sweat, hyperhidrosis" |
| L89 | Decubitus ulcer and pressure area | L89 |  |
| M80 | Osteoporosis with pathological fracture | M80 |  |
| R40 | Somnolence, stupor and coma | R40 | mental change, drowsy, altered metality, semicoma, stupor, coma, solmnolence, Korean word of "altered metality,stupor, coma, solmnolence" |
| R56 | Convulsions, NEC | R56 | i) convulsion, seizure  ii)convulsion, seizure, epilepsy |

Abbreviations: KCD: Korean Standard Classification of Diseases, HIRA: Health Insurance Review and Assessment Service, EMR: Electronic medical records, NEC: not elsewhere classified

**Supplementary Table S3. Baseline characteristics of study subjects in immune checkpoint inhibitors (ICI) use and non-ICI use**

|  |  | ICI | | Non-ICI | | p-value |
| --- | --- | --- | --- | --- | --- | --- |
|  |  | N | (%) | N | (%) |  |
|  |  | 4516 | (100) | 21301 | (100) |  |
| Cancer subtype | Lung | 3751 | (83.1) | 19091 | (89.6) | <.001 |
|  | Melanoma | 513 | (11.4) | 151 | (0.7) |  |
|  | Bladder | 252 | (5.6) | 2059 | (9.7) |  |
| Sex | Male | 3416 | (75.6) | 15718 | (73.8) | <.001 |
|  | Female | 1100 | (24.4) | 5583 | (26.2) |  |
| Age | Mean, SD | 64.94 | (9.97) | 66.67 | (9.99) | <.001 |
| Age group | <30 | 11 | (0.2) | 30 | (0.1) | <.001 |
|  | 30-39 | 35 | (0.8) | 200 | (0.9) |  |
|  | 40-49 | 270 | (6) | 917 | (4.3) |  |
|  | 50-59 | 966 | (21.4) | 3561 | (16.7) |  |
|  | 60-69 | 1656 | (36.7) | 7573 | (35.6) |  |
|  | 70-79 | 1348 | (29.8) | 7381 | (34.7) |  |
|  | 80+ | 230 | (5.1) | 1639 | (7.7) |  |
| Charlson comorbidity index | Mean, SD | 8.56 | (3.59) | 7.23 | (3.59) | <.001 |
|  | <4 | 501 | (11.1) | 3694 | (17.3) | <.001 |
|  | 4~7 | 1030 | (22.8) | 7745 | (36.4) |  |
|  | 8~11 | 2080 | (46.1) | 7106 | (33.4) |  |
|  | ≥12 | 905 | (20) | 2756 | (12.9) |  |

Abbreviations: SD: standard deviation

**Supplementary Table S4. Detected signals of adverse events related to immune checkpoint inhibitors in HIRA database (n=118 types of AE)**

|  |  |  | **ICI user** | | | **Non-ICI-user** | | | | **HR** | | | | | **PCR** | | | | | **COR** | | | | | | | | | **IC**** | | | | |  |  |  |  |  |  |  |  |  |  |  |  |  |  |  |  |  |  |  |  |  |  |  |  |  |  |  |  |  |  |  |  |
| --- | --- | --- | --- | --- | --- | --- | --- | --- | --- | --- | --- | --- | --- | --- | --- | --- | --- | --- | --- | --- | --- | --- | --- | --- | --- | --- | --- | --- | --- | --- | --- | --- | --- | --- | --- | --- | --- | --- | --- | --- | --- | --- | --- | --- | --- | --- | --- | --- | --- | --- | --- | --- | --- | --- | --- | --- | --- | --- | --- | --- | --- | --- | --- | --- | --- |
|  | **KCD code** | **English** | **AE (%)** | **Total** | | **AE (%)** | **Total** | | | **point estimates** | **lower CI** | | **upper CI** | | **point estimates** | **lower CI** | | **upper CI** | | **point estimates** | | **lower CI** | | | **upper CI** | | | |  | | | | |  |  |  |  |  |  |  |  |  |  |  |  |  |  |  |  |  |  |  |  |  |  |  |  |  |  |  |  |  |  |  |  |
|  | **I. Certain infectious and parasitic diseases (A00-B99)** | | | |  | | | | | | | | | | | |  | |  | | | |  | | |  | | | |  | | | | |  | | | |  | | | |  | | | |  | | | |  | | | |  | | | |  | | | |  |  |  |
|  | A30–A49 | Other bacterial diseases | 5.4 | 4,112 | | 5.4 | 20,158 | | | 1.18 | 1.02 | | 1.36 | | 1 | 0.88 | | 1.13 | | 1 | | 0.86 | | | 1.16 | | | | -0.21 | | | | |  |  |  |  |  |  |  |  |  |  |  |  |  |  |  |  |  |  |  |  |  |  |  |  |  |  |  |  |  |  |  |  |
| P* | A41 | Other sepsis | 4.4 | 4,286 | | 4.2 | 20,772 | | | 1.23 | 1.05 | | 1.44 | | 1.04 | 0.91 | | 1.19 | | 1.04 | | 0.89 | | | 1.22 | | | | -0.18 | | | | |  |  |  |  |  |  |  |  |  |  |  |  |  |  |  |  |  |  |  |  |  |  |  |  |  |  |  |  |  |  |  |  |
|  | B00–B09 | Herpes viral infections | 4.8 | 3,475 | | 4.8 | 17,097 | | | 1.23 | 1.05 | | 1.46 | | 1 | 0.87 | | 1.15 | | 1 | | 0.84 | | | 1.18 | | | | -0.24 | | | | |  |  |  |  |  |  |  |  |  |  |  |  |  |  |  |  |  |  |  |  |  |  |  |  |  |  |  |  |  |  |  |  |
| P | B02 | Zoster [herpes zoster] | 3.2 | 3,907 | | 3.1 | 18,874 | | | 1.29 | 1.07 | | 1.57 | | 1.04 | 0.89 | | 1.22 | | 1.04 | | 0.86 | | | 1.27 | | | | -0.22 | | | | |  |  |  |  |  |  |  |  |  |  |  |  |  |  |  |  |  |  |  |  |  |  |  |  |  |  |  |  |  |  |  |  |
|  | B35–B49 | Mycoses | 8.9 | 2,694 | | 8.9 | 13,488 | | | 1.21 | 1.05 | | 1.39 | | 1 | 0.89 | | 1.13 | | 1 | | 0.87 | | | 1.16 | | | | -0.2 | | | | |  |  |  |  |  |  |  |  |  |  |  |  |  |  |  |  |  |  |  |  |  |  |  |  |  |  |  |  |  |  |  |  |
| P | B35 | Dermatophytosis | 4.1 | 3,063 | | 4 | 14,655 | | | 1.3 | 1.08 | | 1.58 | | 1.03 | 0.88 | | 1.21 | | 1.03 | | 0.85 | | | 1.26 | | | | -0.24 | | | | |  |  |  |  |  |  |  |  |  |  |  |  |  |  |  |  |  |  |  |  |  |  |  |  |  |  |  |  |  |  |  |  |
|  | B50–B64 | Protozoal diseases | 0.5 | 4,494 | | 0.4 | 21,272 | | | 1.59 | 1 | | 2.53 | | 1.31 | 0.91 | | 1.88 | | 1.31 | | 0.83 | | | 2.09 | | | | -0.29 | | | | |  |  |  |  |  |  |  |  |  |  |  |  |  |  |  |  |  |  |  |  |  |  |  |  |  |  |  |  |  |  |  |  |
| P | B59 | Pneumocystosis (J17.3) | 0.5 | 4,498 | | 0.4 | 21,289 | | | 1.63 | 1.03 | | 2.59 | | 1.34 | 0.94 | | 1.93 | | 1.35 | | 0.85 | | | 2.14 | | | | -0.26 | | | | |  |  |  |  |  |  |  |  |  |  |  |  |  |  |  |  |  |  |  |  |  |  |  |  |  |  |  |  |  |  |  |  |
|  | **IV. Endocrine, nutritional and metabolic diseases (E00-E90)** | | | | | | |  | | | | | | | | | | | | |  | | |  | | | |  | | | |  | | | | |  | | | |  | | | |  | | | |  | | | |  | | | |  | | | |  | | | |  |
| W* | E00–E07 | Disorders of the thyroid gland | 20.8 | 2,784 | | 7.6 | 14,323 | | | 3.96 | 3.58 | | 4.38 | | 2.73 | 2.52 | | 2.94 | | 3.18 | | 2.85 | | | 3.55 | | | | 0.94 | | | | |  |  |  |  |  |  |  |  |  |  |  |  |  |  |  |  |  |  |  |  |  |  |  |  |  |  |  |  |  |  |  |  |
| W | E02 | Subclinical iodine-deficiency hypothyroidism | 0.2 | 4,506 | | 0.1 | 21,248 | | | 2.84 | 1.22 | | 6.64 | | 2.36 | 1.34 | | 4.15 | | 2.36 | | 1.01 | | | 5.52 | | | | -0.08 | | | | |  |  |  |  |  |  |  |  |  |  |  |  |  |  |  |  |  |  |  |  |  |  |  |  |  |  |  |  |  |  |  |  |
| W | E03 | Other types of hypothyroidism | 5.9 | 4,068 | | 1.7 | 19,313 | | | 4.38 | 3.71 | | 5.17 | | 3.4 | 3.08 | | 3.76 | | 3.55 | | 3 | | | 4.21 | | | | 1.04 | | | | |  |  |  |  |  |  |  |  |  |  |  |  |  |  |  |  |  |  |  |  |  |  |  |  |  |  |  |  |  |  |  |  |
| W | E05 | Thyrotoxicosis [hyperthyroidism] | 1.4 | 4,344 | | 0.5 | 20,454 | | | 3.1 | 2.26 | | 4.25 | | 2.57 | 2.09 | | 3.15 | | 2.59 | | 1.89 | | | 3.55 | | | | 0.59 | | | | |  |  |  |  |  |  |  |  |  |  |  |  |  |  |  |  |  |  |  |  |  |  |  |  |  |  |  |  |  |  |  |  |
| W | E06 | Thyroiditis | 1 | 4,409 | | 0.4 | 20,742 | | | 3.34 | 2.31 | | 4.82 | | 2.71 | 2.15 | | 3.43 | | 2.73 | | 1.89 | | | 3.95 | | | | 0.58 | | | | |  |  |  |  |  |  |  |  |  |  |  |  |  |  |  |  |  |  |  |  |  |  |  |  |  |  |  |  |  |  |  |  |
| W | E07 | Other disorders of thyroid | 16 | 3,280 | | 5.8 | 16,884 | | | 3.8 | 3.42 | | 4.23 | | 2.77 | 2.56 | | 2.99 | | 3.11 | | 2.77 | | | 3.48 | | | | 0.95 | | | | |  |  |  |  |  |  |  |  |  |  |  |  |  |  |  |  |  |  |  |  |  |  |  |  |  |  |  |  |  |  |  |  |
| W | E10–E14 | Diabetes mellitus | 10.7 | 2,195 | | 10.5 | 10,637 | | | 1.22 | 1.06 | | 1.4 | | 1.02 | 0.9 | | 1.16 | | 1.03 | | 0.88 | | | 1.19 | | | | -0.18 | | | | |  |  |  |  |  |  |  |  |  |  |  |  |  |  |  |  |  |  |  |  |  |  |  |  |  |  |  |  |  |  |  |  |
| P | E11 | Type 2 diabetes mellitus | 9 | 2,535 | | 8.7 | 12,031 | | | 1.22 | 1.06 | | 1.41 | | 1.03 | 0.91 | | 1.17 | | 1.04 | | 0.89 | | | 1.21 | | | | -0.17 | | | | |  |  |  |  |  |  |  |  |  |  |  |  |  |  |  |  |  |  |  |  |  |  |  |  |  |  |  |  |  |  |  |  |
|  | E20–E35 | Disorders of other endocrine glands | 3.7 | 4,224 | | 2.6 | 20,126 | | | 1.69 | 1.41 | | 2.02 | | 1.39 | 1.21 | | 1.6 | | 1.41 | | 1.17 | | | 1.69 | | | | 0.13 | | | | |  |  |  |  |  |  |  |  |  |  |  |  |  |  |  |  |  |  |  |  |  |  |  |  |  |  |  |  |  |  |  |  |
| P | E24 | Cushing’s syndrome | 0.1 | 4,509 | | 0 | 21,262 | | | 5.74 | 1.66 | | 19.85 | | 4.72 | 2.54 | | 8.77 | | 4.72 | | 1.37 | | | 16.31 | | | | 0.18 | | | | |  |  |  |  |  |  |  |  |  |  |  |  |  |  |  |  |  |  |  |  |  |  |  |  |  |  |  |  |  |  |  |  |
| P | E27 | Other disorders of the adrenal gland | 2.2 | 4,363 | | 1.5 | 20,945 | | | 1.79 | 1.42 | | 2.24 | | 1.49 | 1.25 | | 1.77 | | 1.5 | | 1.19 | | | 1.89 | | | | 0.14 | | | | |  |  |  |  |  |  |  |  |  |  |  |  |  |  |  |  |  |  |  |  |  |  |  |  |  |  |  |  |  |  |  |  |
|  | E35 | Disorders of the endocrine glands in diseases classified elsewhere | 0.5 | 4,501 | | 0.1 | 21,196 | | | 5.54 | 3.16 | | 9.71 | | 4.52 | 3.39 | | 6.02 | | 4.54 | | 2.59 | | | 7.96 | | | | 0.81 | | | | |  |  |  |  |  |  |  |  |  |  |  |  |  |  |  |  |  |  |  |  |  |  |  |  |  |  |  |  |  |  |  |  |
| P | E83 | Disorders of mineral metabolism | 4.5 | 4,191 | | 3.4 | 20,206 | | | 1.58 | 1.34 | | 1.86 | | 1.33 | 1.17 | | 1.51 | | 1.34 | | 1.14 | | | 1.58 | | | | 0.1 | | | | |  |  |  |  |  |  |  |  |  |  |  |  |  |  |  |  |  |  |  |  |  |  |  |  |  |  |  |  |  |  |  |  |
|  | E89 | Postprocedural endocrine and metabolic disorders, NEC | 0.4 | 4,467 | | 0.2 | 21,124 | | | 2.2 | 1.23 | | 3.91 | | 1.8 | 1.19 | | 2.74 | | 1.8 | | 1.01 | | | 3.21 | | | | -0.07 | | | | |  |  |  |  |  |  |  |  |  |  |  |  |  |  |  |  |  |  |  |  |  |  |  |  |  |  |  |  |  |  |  |  |
|  | **V. Mental and behavioural disorders (F00-F99)** | | | |  | | | | | | | | | | | |  | |  | | | |  | | |  | | | |  | | | | |  | | | |  | | | |  | | | |  | | | |  | | | |  | | | |  | | | |  |  |  |
|  | F00–F09 | Organic, including symptomatic, mental disorders | 6.8† | 3,905 | | 7 | 18,294 | | | 1.17 | 1.02 | | 1.33 | | 0.97 | 0.87 | | 1.09 | | 0.97 | | 0.84 | | | 1.11 | | | | -0.23 | | | | |  |  |  |  |  |  |  |  |  |  |  |  |  |  |  |  |  |  |  |  |  |  |  |  |  |  |  |  |  |  |  |  |
| P | F05 | Delirium, not induced by alcohol and other psychoactive substances | 3.9 | 4,409 | | 3.2 | 21,017 | | | 1.48 | 1.25 | | 1.75 | | 1.23 | 1.08 | | 1.41 | | 1.24 | | 1.05 | | | 1.47 | | | | 0.01 | | | | |  |  |  |  |  |  |  |  |  |  |  |  |  |  |  |  |  |  |  |  |  |  |  |  |  |  |  |  |  |  |  |  |
|  | F29 | Unspecified nonorganic psychosis | 0.4 | 4,479 | | 0.2 | 21,234 | | | 1.78 | 1.02 | | 3.12 | | 1.49 | 0.97 | | 2.28 | | 1.49 | | 0.85 | | | 2.61 | | | | -0.26 | | | | |  |  |  |  |  |  |  |  |  |  |  |  |  |  |  |  |  |  |  |  |  |  |  |  |  |  |  |  |  |  |  |  |
| P | F30–F39 | Mood [affective] disorders | 10.9† | 3,128 | | 11.2 | 15,855 | | | 1.14 | 1.02 | | 1.29 | | 0.97 | 0.88 | | 1.08 | | 0.97 | | 0.86 | | | 1.09 | | | | -0.21 | | | | |  |  |  |  |  |  |  |  |  |  |  |  |  |  |  |  |  |  |  |  |  |  |  |  |  |  |  |  |  |  |  |  |
|  | F31 | Bipolar affective disorder | 4.7† | 4,229 | | 4.8 | 20,449 | | | 1.17 | 1.01 | | 1.37 | | 0.98 | 0.86 | | 1.12 | | 0.98 | | 0.84 | | | 1.15 | | | | -0.24 | | | | |  |  |  |  |  |  |  |  |  |  |  |  |  |  |  |  |  |  |  |  |  |  |  |  |  |  |  |  |  |  |  |  |
|  | F41 | Other anxiety disorders | 8.4† | 2,935 | | 8.6 | 14,638 | | | 1.16 | 1.01 | | 1.33 | | 0.98 | 0.87 | | 1.1 | | 0.98 | | 0.85 | | | 1.13 | | | | -0.22 | | | | |  |  |  |  |  |  |  |  |  |  |  |  |  |  |  |  |  |  |  |  |  |  |  |  |  |  |  |  |  |  |  |  |
|  | **VI. Diseases of the nervous system (G00-G99)** | | | |  | | | | | | | | | | | |  | |  | | | |  | | |  | | | |  | | | | |  | | | |  | | | |  | | | |  | | | |  | | | |  | | | |  | | | |  |  |  |
| W | G03 | Meningitis due to other and unspecified causes | 0.1 | 4,512 | | 0 | 21,283 | | | 3.27 | 0.78 | | 13.71 | | 2.83 | 1.16 | | 6.93 | | 2.83 | | 0.68 | | | 11.85 | | | | -0.39 | | | | |  |  |  |  |  |  |  |  |  |  |  |  |  |  |  |  |  |  |  |  |  |  |  |  |  |  |  |  |  |  |  |  |
|  | G32 | Other degenerative disorders of the nervous system in diseases classified elsewhere | 0.1 | 4,516 | | 0 | 21,283 | | | 3.1 | 1.04 | | 9.27 | | 2.62 | 1.3 | | 5.29 | | 2.62 | | 0.88 | | | 7.82 | | | | -0.02 | | | | |  |  |  |  |  |  |  |  |  |  |  |  |  |  |  |  |  |  |  |  |  |  |  |  |  |  |  |  |  |  |  |  |
| P | G40 | Epilepsy | 3.8 | 4,107 | | 3 | 20,088 | | | 1.58 | 1.33 | | 1.88 | | 1.29 | 1.12 | | 1.49 | | 1.3 | | 1.09 | | | 1.56 | | | | 0.05 | | | | |  |  |  |  |  |  |  |  |  |  |  |  |  |  |  |  |  |  |  |  |  |  |  |  |  |  |  |  |  |  |  |  |
| W | G50–G59 | Nerve, nerve root, and plexus disorders | 3.6 | 3,483 | | 3.3 | 16,706 | | | 1.33 | 1.1 | | 1.62 | | 1.08 | 0.92 | | 1.27 | | 1.09 | | 0.89 | | | 1.32 | | | | -0.18 | | | | |  |  |  |  |  |  |  |  |  |  |  |  |  |  |  |  |  |  |  |  |  |  |  |  |  |  |  |  |  |  |  |  |
| W | G51 | Facial nerve disorders | 0.3 | 4,461 | | 0.2 | 21,023 | | | 1.9 | 1.01 | | 3.55 | | 1.57 | 0.98 | | 2.52 | | 1.57 | | 0.84 | | | 2.95 | | | | -0.27 | | | | |  |  |  |  |  |  |  |  |  |  |  |  |  |  |  |  |  |  |  |  |  |  |  |  |  |  |  |  |  |  |  |  |
| W | G53 | Cranial nerve disorders in diseases classified elsewhere | 2 | 4,150 | | 1.8 | 19,733 | | | 1.39 | 1.09 | | 1.77 | | 1.13 | 0.93 | | 1.37 | | 1.13 | | 0.89 | | | 1.44 | | | | -0.19 | | | | |  |  |  |  |  |  |  |  |  |  |  |  |  |  |  |  |  |  |  |  |  |  |  |  |  |  |  |  |  |  |  |  |
| W | G70–G73 | Diseases of the myoneural junction and muscle | 0.2 | 4,494 | | 0.1 | 21,195 | | | 2.68 | 1.09 | | 6.57 | | 2.2 | 1.19 | | 4.06 | | 2.2 | | 0.9 | | | 5.41 | | | | -0.19 | | | | |  |  |  |  |  |  |  |  |  |  |  |  |  |  |  |  |  |  |  |  |  |  |  |  |  |  |  |  |  |  |  |  |
|  | G80–G83 | Cerebral palsy and other paralytic syndromes | 2.2 | 4,362 | | 1.5 | 20,811 | | | 1.77 | 1.4 | | 2.22 | | 1.46 | 1.22 | | 1.74 | | 1.47 | | 1.17 | | | 1.85 | | | | 0.12 | | | | |  |  |  |  |  |  |  |  |  |  |  |  |  |  |  |  |  |  |  |  |  |  |  |  |  |  |  |  |  |  |  |  |
| P | G81 | Hemiplegia | 1.6 | 4,420 | | 1 | 20,971 | | | 1.94 | 1.47 | | 2.55 | | 1.6 | 1.31 | | 1.97 | | 1.61 | | 1.23 | | | 2.13 | | | | 0.16 | | | | |  |  |  |  |  |  |  |  |  |  |  |  |  |  |  |  |  |  |  |  |  |  |  |  |  |  |  |  |  |  |  |  |
| P | G83 | Other paralytic syndromes | 0.3 | 4,482 | | 0.1 | 21,187 | | | 2.65 | 1.34 | | 5.26 | | 2.18 | 1.37 | | 3.49 | | 2.18 | | 1.1 | | | 4.33 | | | | 0.01 | | | | |  |  |  |  |  |  |  |  |  |  |  |  |  |  |  |  |  |  |  |  |  |  |  |  |  |  |  |  |  |  |  |  |
|  | G90–G99 | Other disorders of the nervous system | 1.7 | 4,266 | | 1.4 | 20,221 | | | 1.49 | 1.15 | | 1.92 | | 1.23 | 1 | | 1.51 | | 1.23 | | 0.95 | | | 1.59 | | | | -0.11 | | | | |  |  |  |  |  |  |  |  |  |  |  |  |  |  |  |  |  |  |  |  |  |  |  |  |  |  |  |  |  |  |  |  |
|  | G93 | Other disorders of the brain | 0.8 | 4,449 | | 0.5 | 21,015 | | | 1.93 | 1.32 | | 2.83 | | 1.59 | 1.19 | | 2.12 | | 1.59 | | 1.09 | | | 2.34 | | | | 0.02 | | | | |  |  |  |  |  |  |  |  |  |  |  |  |  |  |  |  |  |  |  |  |  |  |  |  |  |  |  |  |  |  |  |  |
|  | **VII. Diseases of the eye and adnexa (H00-H59)** | | | |  | | | | | | | | | | | |  | |  | | | |  | | |  | | | |  | | | | |  | | | |  | | | |  | | | |  | | | |  | | | |  | | | |  | | | |  |  |  |
| W | H20 | Iridocyclitis | 0.3 | 4,401 | | 0.2 | 20,749 | | | 1.87 | 1.05 | | 3.35 | | 1.5 | 0.97 | | 2.34 | | 1.51 | | 0.84 | | | 2.70 | | | | -0.27 | | | | |  |  |  |  |  |  |  |  |  |  |  |  |  |  |  |  |  |  |  |  |  |  |  |  |  |  |  |  |  |  |  |  |
| P | H28 | Cataract and other disorders of lens in diseases classified elsewhere | 0.1 | 4,514 | | 0 | 21,256 | | | 5.82 | 1.45 | | 23.27 | | 4.71 | 2.35 | | 9.42 | | 4.71 | | 1.18 | | | 18.85 | | | | 0.06 | | | | |  |  |  |  |  |  |  |  |  |  |  |  |  |  |  |  |  |  |  |  |  |  |  |  |  |  |  |  |  |  |  |  |
| P | H30 | Chorioretinal inflammation | 0.1 | 4,496 | | 0.1 | 21,203 | | | 2.43 | 0.93 | | 6.33 | | 2.02 | 1.03 | | 3.95 | | 2.02 | | 0.78 | | | 5.27 | | | | -0.33 | | | | |  |  |  |  |  |  |  |  |  |  |  |  |  |  |  |  |  |  |  |  |  |  |  |  |  |  |  |  |  |  |  |  |
|  | **VIII. Diseases of the ear and mastoid process (H60-H95)** | | | | | | |  | | | | | | | | | | | | |  | | |  | | | |  | | | |  | | | | |  | | | |  | | | |  | | | |  | | | |  | | | |  | | | |  | | | |  |
|  | H61 | Other disorders of the external ear | 1.2 | 4,166 | | 1.1 | 19,593 | | | 1.4 | 1.03 | | 1.9 | | 1.13 | 0.88 | | 1.44 | | 1.13 | | 0.83 | | | 1.53 | | | | -0.28 | | | | |  |  |  |  |  |  |  |  |  |  |  |  |  |  |  |  |  |  |  |  |  |  |  |  |  |  |  |  |  |  |  |  |
| P | H67 | Otitis media in diseases classified elsewhere | 0.1 | 4,511 | | 0 | 21,271 | | | 5.94 | 1.2 | | 29.41 | | 4.72 | 2.12 | | 10.5 | | 4.72 | | 0.95 | | | 23.38 | | | | -0.1 | | | | |  |  |  |  |  |  |  |  |  |  |  |  |  |  |  |  |  |  |  |  |  |  |  |  |  |  |  |  |  |  |  |  |
|  | **IX. Diseases of the circulatory system (I00-I99)** | | | |  | | | | | | | | | | | |  | |  | | | |  | | |  | | | |  | | | | |  | | | |  | | | |  | | | |  | | | |  | | | |  | | | |  | | | |  |  |  |
|  | I30–I52 | Other forms of heart disease | 12.4 | 3,279 | | 11 | 16,265 | | | 1.32 | 1.19 | | 1.47 | | 1.12 | 1.02 | | 1.23 | | 1.14 | | 1.02 | | | 1.28 | | | | -0.02 | | | | |  |  |  |  |  |  |  |  |  |  |  |  |  |  |  |  |  |  |  |  |  |  |  |  |  |  |  |  |  |  |  |  |
|  | I31 | Other diseases of the pericardium | 1 | 4,375 | | 0.8 | 21,012 | | | 1.65 | 1.18 | | 2.29 | | 1.37 | 1.06 | | 1.77 | | 1.37 | | 0.98 | | | 1.91 | | | | -0.09 | | | | |  |  |  |  |  |  |  |  |  |  |  |  |  |  |  |  |  |  |  |  |  |  |  |  |  |  |  |  |  |  |  |  |
| P | I46 | Cardiac arrest | 4.4 | 4,490 | | 2.7 | 21,243 | | | 1.97 | 1.67 | | 2.31 | | 1.64 | 1.45 | | 1.85 | | 1.67 | | 1.41 | | | 1.97 | | | | 0.33 | | | | |  |  |  |  |  |  |  |  |  |  |  |  |  |  |  |  |  |  |  |  |  |  |  |  |  |  |  |  |  |  |  |  |
| P | I47 | Paroxysmal tachycardia | 0.9 | 4,442 | | 0.6 | 21,016 | | | 1.64 | 1.14 | | 2.36 | | 1.36 | 1.03 | | 1.8 | | 1.37 | | 0.95 | | | 1.96 | | | | -0.13 | | | | |  |  |  |  |  |  |  |  |  |  |  |  |  |  |  |  |  |  |  |  |  |  |  |  |  |  |  |  |  |  |  |  |
| P | I61 | Intracerebral hemorrhage | 0.4 | 4,480 | | 0.3 | 21,137 | | | 1.96 | 1.18 | | 3.25 | | 1.63 | 1.11 | | 2.38 | | 1.63 | | 0.98 | | | 2.71 | | | | -0.11 | | | | |  |  |  |  |  |  |  |  |  |  |  |  |  |  |  |  |  |  |  |  |  |  |  |  |  |  |  |  |  |  |  |  |
| P | I81 | Portal vein thrombosis | 0.1 | 4,515 | | 0 | 21,291 | | | 5.57 | 1.79 | | 17.28 | | 4.72 | 2.68 | | 8.31 | | 4.72 | | 1.52 | | | 14.64 | | | | 0.27 | | | | |  |  |  |  |  |  |  |  |  |  |  |  |  |  |  |  |  |  |  |  |  |  |  |  |  |  |  |  |  |  |  |  |
|  | I89 | Other noninfective disorders of the lymphatic vessels and lymph nodes | 1.5 | 4,243 | | 1.3 | 20,545 | | | 1.33 | 1.01 | | 1.75 | | 1.1 | 0.88 | | 1.38 | | 1.1 | | 0.84 | | | 1.45 | | | | -0.26 | | | | |  |  |  |  |  |  |  |  |  |  |  |  |  |  |  |  |  |  |  |  |  |  |  |  |  |  |  |  |  |  |  |  |
|  | **X. Diseases of the respiratory system (J00-J99)** | | | |  | | | | | | | | | | | |  | |  | | | |  | | |  | | | |  | | | | |  | | | |  | | | |  | | | |  | | | |  | | | |  | | | |  | | | |  |  |  |
|  | J09–J18 | Influenza and pneumonia | 21.1 | 2,203 | | 21.3 | 12,463 | | | 1.16 | 1.05 | | 1.28 | | 0.99 | 0.9 | | 1.09 | | 0.99 | | 0.88 | | | 1.10 | | | | -0.17 | | | | |  |  |  |  |  |  |  |  |  |  |  |  |  |  |  |  |  |  |  |  |  |  |  |  |  |  |  |  |  |  |  |  |
|  | J15 | Bacterial pneumonia, NEC | 3 | 3,989 | | 2.8 | 19,099 | | | 1.3 | 1.07 | | 1.58 | | 1.08 | 0.92 | | 1.27 | | 1.08 | | 0.89 | | | 1.32 | | | | -0.19 | | | | |  |  |  |  |  |  |  |  |  |  |  |  |  |  |  |  |  |  |  |  |  |  |  |  |  |  |  |  |  |  |  |  |
| W | J18 | Pneumonia, organism unspecified | 18.6† | 2,566 | | 18.8 | 14,163 | | | 1.16 | 1.05 | | 1.28 | | 0.99 | 0.9 | | 1.09 | | 0.99 | | 0.89 | | | 1.10 | | | | -0.16 | | | | |  |  |  |  |  |  |  |  |  |  |  |  |  |  |  |  |  |  |  |  |  |  |  |  |  |  |  |  |  |  |  |  |
| P | J46 | Status asthmaticus | 1.6 | 4,245 | | 1.4 | 20,219 | | | 1.32 | 1.01 | | 1.72 | | 1.08 | 0.87 | | 1.35 | | 1.09 | | 0.83 | | | 1.42 | | | | -0.27 | | | | |  |  |  |  |  |  |  |  |  |  |  |  |  |  |  |  |  |  |  |  |  |  |  |  |  |  |  |  |  |  |  |  |
| P | J67 | Hypersensitivity pneumonitis due to organic dust | 0.1 | 4,514 | | 0 | 21,295 | | | 4.44 | 1.19 | | 16.54 | | 3.77 | 1.82 | | 7.84 | | 3.78 | | 1.01 | | | 14.07. | | | | -0.07 | | | | |  |  |  |  |  |  |  |  |  |  |  |  |  |  |  |  |  |  |  |  |  |  |  |  |  |  |  |  |  |  |  |  |
|  | J90–J94 | Other diseases of pleura | 9.3 | 3,501 | | 7.1 | 17,681 | | | 1.53 | 1.36 | | 1.73 | | 1.3 | 1.17 | | 1.43 | | 1.33 | | 1.17 | | | 1.51 | | | | 0.13 | | | | |  |  |  |  |  |  |  |  |  |  |  |  |  |  |  |  |  |  |  |  |  |  |  |  |  |  |  |  |  |  |  |  |
| W | J90 | Pleural effusion, NEC | 7.2 | 3,706 | | 5.5 | 18,540 | | | 1.55 | 1.35 | | 1.77 | | 1.3 | 1.17 | | 1.46 | | 1.33 | | 1.15 | | | 1.53 | | | | 0.12 | | | | |  |  |  |  |  |  |  |  |  |  |  |  |  |  |  |  |  |  |  |  |  |  |  |  |  |  |  |  |  |  |  |  |
|  | J91 | Pleural effusion in conditions classified elsewhere | 1.9 | 4,369 | | 1.3 | 20,865 | | | 1.72 | 1.34 | | 2.21 | | 1.45 | 1.19 | | 1.76 | | 1.46 | | 1.13 | | | 1.87 | | | | 0.08 | | | | |  |  |  |  |  |  |  |  |  |  |  |  |  |  |  |  |  |  |  |  |  |  |  |  |  |  |  |  |  |  |  |  |
| W | J96 | Respiratory failure, NEC | 2.5 | 4,442 | | 2.1 | 21,086 | | | 1.42 | 1.15 | | 1.74 | | 1.17 | 0.99 | | 1.39 | | 1.18 | | 0.95 | | | 1.45 | | | | -0.11 | | | | |  |  |  |  |  |  |  |  |  |  |  |  |  |  |  |  |  |  |  |  |  |  |  |  |  |  |  |  |  |  |  |  |
|  | **XI. Diseases of the digestive system (K00-K93)** | | | |  | | | | | | | | | | | |  | |  | | | |  | | |  | | | |  | | | | |  | | | |  | | | |  | | | |  | | | |  | | | |  | | | |  | | | |  |  |  |
|  | K35–K38 | Diseases of appendix | 0.2 | 4,469 | | 0.1 | 21,095 | | | 2.04 | 1.02 | | 4.06 | | 1.67 | 1.01 | | 2.78 | | 1.68 | | 0.84 | | | 3.34 | | | | -0.27 | | | | |  |  |  |  |  |  |  |  |  |  |  |  |  |  |  |  |  |  |  |  |  |  |  |  |  |  |  |  |  |  |  |  |
| P | K35 | Acute appendicitis | 0.2 | 4,475 | | 0.1 | 21,131 | | | 2.35 | 1.08 | | 5.11 | | 1.93 | 1.11 | | 3.35 | | 1.93 | | 0.89 | | | 4.20 | | | | -0.21 | | | | |  |  |  |  |  |  |  |  |  |  |  |  |  |  |  |  |  |  |  |  |  |  |  |  |  |  |  |  |  |  |  |  |
|  | K65–K67 | Diseases of peritoneum | 0.5 | 4471 | | 0.3 | 21,133 | | | 1.82 | 1.14 | | 2.92 | | 1.51 | 1.06 | | 2.16 | | 1.51 | | 0.94 | | | 2.42 | | | | -0.14 | | | | |  |  |  |  |  |  |  |  |  |  |  |  |  |  |  |  |  |  |  |  |  |  |  |  |  |  |  |  |  |  |  |  |
|  | K66 | Other disorders of the peritoneum | 0.1 | 4,505 | | 0.1 | 21,255 | | | 2.65 | 1.01 | | 6.97 | | 2.18 | 1.12 | | 4.22 | | 2.18 | | 0.83 | | | 5.74 | | | | -0.27 | | | | |  |  |  |  |  |  |  |  |  |  |  |  |  |  |  |  |  |  |  |  |  |  |  |  |  |  |  |  |  |  |  |  |
| W | K71 | Toxic liver disease | 4 | 4,142 | | 3.7 | 20,034 | | | 1.26 | 1.06 | | 1.49 | | 1.07 | 0.93 | | 1.24 | | 1.08 | | 0.91 | | | 1.28 | | | | -0.16 | | | | |  |  |  |  |  |  |  |  |  |  |  |  |  |  |  |  |  |  |  |  |  |  |  |  |  |  |  |  |  |  |  |  |
| W | K73 | Chronic hepatitis, NEC | 3.2 | 4,096 | | 3 | 19,393 | | | 1.29 | 1.07 | | 1.55 | | 1.08 | 0.93 | | 1.27 | | 1.09 | | 0.9 | | | 1.32 | | | | -0.17 | | | | |  |  |  |  |  |  |  |  |  |  |  |  |  |  |  |  |  |  |  |  |  |  |  |  |  |  |  |  |  |  |  |  |
|  | K80–K87 | Disorders of the gallbladder, biliary tract, and pancreas | 5.7 | 3,663 | | 5.4 | 17,731 | | | 1.26 | 1.09 | | 1.47 | | 1.06 | 0.93 | | 1.2 | | 1.06 | | 0.91 | | | 1.24 | | | | -0.15 | | | | |  |  |  |  |  |  |  |  |  |  |  |  |  |  |  |  |  |  |  |  |  |  |  |  |  |  |  |  |  |  |  |  |
|  | K83 | Other diseases of biliary tract | 1 | 4,397 | | 0.8 | 20,806 | | | 1.5 | 1.08 | | 2.08 | | 1.25 | 0.97 | | 1.62 | | 1.25 | | 0.9 | | | 1.74 | | | | -0.18 | | | | |  |  |  |  |  |  |  |  |  |  |  |  |  |  |  |  |  |  |  |  |  |  |  |  |  |  |  |  |  |  |  |  |
|  | K86 | Other diseases of pancreas | 2.3 | 4,213 | | 2.1 | 19,900 | | | 1.3 | 1.04 | | 1.63 | | 1.08 | 0.9 | | 1.3 | | 1.08 | | 0.87 | | | 1.36 | | | | -0.22 | | | | |  |  |  |  |  |  |  |  |  |  |  |  |  |  |  |  |  |  |  |  |  |  |  |  |  |  |  |  |  |  |  |  |
|  | K87 | Disorders of gallbladder, biliary tract, and pancreas in diseases classified elsewhere | 0.4 | 4,494 | | 0.1 | 21,253 | | | 5.34 | 2.75 | | 10.37 | | 4.47 | 3.17 | | 6.29 | | 4.48 | | 2.31 | | | 8.70 | | | | 0.68 | | | | |  |  |  |  |  |  |  |  |  |  |  |  |  |  |  |  |  |  |  |  |  |  |  |  |  |  |  |  |  |  |  |  |
|  | **XII Diseases of the skin and subcutaneous tissue (L00-L99)** | | | | | | |  | | | | | | | | | | | | |  | | |  | | | |  | | | |  | | | | |  | | | |  | | | |  | | | |  | | | |  | | | |  | | | |  | | | |  |
| W | L20 | Atopic dermatitis | 4.6 | 3,880 | | 3.9 | 18,914 | | | 1.42 | 1.21 | | 1.68 | | 1.18 | 1.03 | | 1.35 | | 1.19 | | 1.01 | | | 1.41 | | | | -0.04 | | | | |  |  |  |  |  |  |  |  |  |  |  |  |  |  |  |  |  |  |  |  |  |  |  |  |  |  |  |  |  |  |  |  |
| W | L23 | Allergic contact dermatitis | 7.7† | 2,326 | | 7.8 | 11,260 | | | 1.2 | 1.02 | | 1.41 | | 0.98 | 0.85 | | 1.12 | | 0.98 | | 0.83 | | | 1.15 | | | | -0.26 | | | | |  |  |  |  |  |  |  |  |  |  |  |  |  |  |  |  |  |  |  |  |  |  |  |  |  |  |  |  |  |  |  |  |
| W | L25 | Unspecified contact dermatitis | 1.9 | 4,042 | | 1.7 | 19,192 | | | 1.37 | 1.07 | | 1.76 | | 1.12 | 0.91 | | 1.36 | | 1.12 | | 0.87 | | | 1.43 | | | | -0.21 | | | | |  |  |  |  |  |  |  |  |  |  |  |  |  |  |  |  |  |  |  |  |  |  |  |  |  |  |  |  |  |  |  |  |
| W | L27 | Dermatitis due to substances taken internally | 3 | 4,151 | | 2.7 | 20,665 | | | 1.32 | 1.09 | | 1.6 | | 1.13 | 0.96 | | 1.32 | | 1.13 | | 0.93 | | | 1.38 | | | | -0.13 | | | | |  |  |  |  |  |  |  |  |  |  |  |  |  |  |  |  |  |  |  |  |  |  |  |  |  |  |  |  |  |  |  |  |
| W | L28 | Lichen simplex chronicus and prurigo | 2.5 | 4,080 | | 2.1 | 19,371 | | | 1.39 | 1.12 | | 1.73 | | 1.14 | 0.96 | | 1.37 | | 1.15 | | 0.92 | | | 1.43 | | | | -0.15 | | | | |  |  |  |  |  |  |  |  |  |  |  |  |  |  |  |  |  |  |  |  |  |  |  |  |  |  |  |  |  |  |  |  |
| W | L29 | Pruritus | 11.4† | 2,916 | | 11.9 | 15,391 | | | 1.16 | 1.04 | | 1.31 | | 0.96 | 0.87 | | 1.07 | | 0.96 | | 0.85 | | | 1.09 | | | | -0.22 | | | | |  |  |  |  |  |  |  |  |  |  |  |  |  |  |  |  |  |  |  |  |  |  |  |  |  |  |  |  |  |  |  |  |
| W | L40–L45 | Papulosquamous disorders | 1.1 | 4,287 | | 0.9 | 20,375 | | | 1.4 | 1.01 | | 1.92 | | 1.16 | 0.9 | | 1.51 | | 1.17 | | 0.85 | | | 1.61 | | | | -0.26 | | | | |  |  |  |  |  |  |  |  |  |  |  |  |  |  |  |  |  |  |  |  |  |  |  |  |  |  |  |  |  |  |  |  |
| W | L43 | Lichen planus | 0.1 | 4,501 | | 0 | 21,245 | | | 11.48 | 2.87 | | 45.95 | | 9.44 | 5.94 | | 14.99 | | 9.45 | | 2.36 | | | 37.81 | | | | 0.58 | | | | |  |  |  |  |  |  |  |  |  |  |  |  |  |  |  |  |  |  |  |  |  |  |  |  |  |  |  |  |  |  |  |  |
| W | L51 | Erythema multiforme | 0.2 | 4,497 | | 0 | 21,249 | | | 5.57 | 2.32 | | 13.39 | | 4.73 | 3.05 | | 7.33 | | 4.73 | | 1.97 | | | 11.38 | | | | 0.51 | | | | |  |  |  |  |  |  |  |  |  |  |  |  |  |  |  |  |  |  |  |  |  |  |  |  |  |  |  |  |  |  |  |  |
|  | L53 | Other erythematous conditions | 0.3 | 4,484 | | 0.2 | 21,165 | | | 2.1 | 1.08 | | 4.08 | | 1.77 | 1.09 | | 2.87 | | 1.77 | | 0.91 | | | 3.44 | | | | -0.19 | | | | |  |  |  |  |  |  |  |  |  |  |  |  |  |  |  |  |  |  |  |  |  |  |  |  |  |  |  |  |  |  |  |  |
| P | L74 | Eccrine sweat disorders | 0.1 | 4,506 | | 0 | 21,239 | | | 5.7 | 1.65 | | 19.71 | | 4.71 | 2.53 | | 8.77 | | 4.72 | | 1.37 | | | 16.30 | | | | 0.17 | | | | |  |  |  |  |  |  |  |  |  |  |  |  |  |  |  |  |  |  |  |  |  |  |  |  |  |  |  |  |  |  |  |  |
|  | L80–L99 | Other disorders of the skin and subcutaneous tissue | 11 | 3,633 | | 8.3 | 18,038 | | | 1.58 | 1.42 | | 1.77 | | 1.33 | 1.21 | | 1.46 | | 1.37 | | 1.22 | | | 1.54 | | | | 0.17 | | | | |  |  |  |  |  |  |  |  |  |  |  |  |  |  |  |  |  |  |  |  |  |  |  |  |  |  |  |  |  |  |  |  |
| W | L80 | Vitiligo | 0.3 | 4,484 | | 0 | 21,212 | | | 17.66 | 5.69 | | 54.79 | | 14.19 | 10.68 | | 18.86 | | 14.23 | | 4.59 | | | 44.13 | | | | 1.07 | | | | |  |  |  |  |  |  |  |  |  |  |  |  |  |  |  |  |  |  |  |  |  |  |  |  |  |  |  |  |  |  |  |  |
| P | L89 | Decubitus ulcer and pressure area | 8.8 | 4,305 | | 6.2 | 20,806 | | | 1.68 | 1.5 | | 1.88 | | 1.41 | 1.29 | | 1.55 | | 1.45 | | 1.29 | | | 1.63 | | | | 0.23 | | | | |  |  |  |  |  |  |  |  |  |  |  |  |  |  |  |  |  |  |  |  |  |  |  |  |  |  |  |  |  |  |  |  |
|  | **XIII. Diseases of the musculoskeletal system and connective tissue (M00-M99)** | | | | | | | |  | | | | | | | | | | | | | |  | | |  | | | |  | | | | |  | | | |  | | | |  | | | |  | | | |  | | | |  | | | |  | | | |  |  |  |
|  | M00–M25 | Arthropathies | 17.5† | 1,024 | | 17.8 | 5,166 | | | 1.18 | 1 | | 1.38 | | 0.98 | 0.85 | | 1.14 | | 0.98 | | 0.82 | | | 1.16 | | | | -0.26 | | | | |  |  |  |  |  |  |  |  |  |  |  |  |  |  |  |  |  |  |  |  |  |  |  |  |  |  |  |  |  |  |  |  |
|  | M25 | Other joint disorders, NEC | 6.4† | 2,839 | | 6.7 | 13,589 | | | 1.2 | 1.02 | | 1.41 | | 0.95 | 0.83 | | 1.09 | | 0.95 | | 0.81 | | | 1.12 | | | | -0.29 | | | | |  |  |  |  |  |  |  |  |  |  |  |  |  |  |  |  |  |  |  |  |  |  |  |  |  |  |  |  |  |  |  |  |
|  | M30–M36 | Systemic connective tissue disorders | 0.5 | 4,439 | | 0.3 | 20,965 | | | 2.39 | 1.47 | | 3.89 | | 1.94 | 1.37 | | 2.74 | | 1.94 | | 1.2 | | | 3.16 | | | | 0.11 | | | | |  |  |  |  |  |  |  |  |  |  |  |  |  |  |  |  |  |  |  |  |  |  |  |  |  |  |  |  |  |  |  |  |
| W | M32 | Systemic lupus erythematosus | 0.1 | 4,502 | | 0 | 21,219 | | | 4.92 | 1.65 | | 14.65 | | 4.04 | 2.24 | | 7.27 | | 4.04 | | 1.36 | | | 12.04 | | | | 0.18 | | | | |  |  |  |  |  |  |  |  |  |  |  |  |  |  |  |  |  |  |  |  |  |  |  |  |  |  |  |  |  |  |  |  |
| W | M35 | Other systemic involvement of connective tissue | 0.4 | 4,463 | | 0.2 | 21,090 | | | 2.77 | 1.53 | | 5.01 | | 2.22 | 1.48 | | 3.33 | | 2.23 | | 1.23 | | | 4.04 | | | | 0.12 | | | | |  |  |  |  |  |  |  |  |  |  |  |  |  |  |  |  |  |  |  |  |  |  |  |  |  |  |  |  |  |  |  |  |
|  | M80–M94 | Osteopathies and chondropathies | 3.9 | 3,461 | | 3.7 | 15,936 | | | 1.3 | 1.07 | | 1.56 | | 1.05 | 0.9 | | 1.23 | | 1.05 | | 0.87 | | | 1.28 | | | | -0.21 | | | | |  |  |  |  |  |  |  |  |  |  |  |  |  |  |  |  |  |  |  |  |  |  |  |  |  |  |  |  |  |  |  |  |
|  | M80–M85 | Disorders of bone density and structure | 3.5 | 3,590 | | 3.2 | 16,439 | | | 1.34 | 1.1 | | 1.62 | | 1.09 | 0.93 | | 1.28 | | 1.09 | | 0.89 | | | 1.33 | | | | -0.18 | | | | |  |  |  |  |  |  |  |  |  |  |  |  |  |  |  |  |  |  |  |  |  |  |  |  |  |  |  |  |  |  |  |  |
| P | M80 | Osteoporosis with pathological fracture | 0.7 | 4,437 | | 0.5 | 20,853 | | | 1.89 | 1.27 | | 2.82 | | 1.55 | 1.15 | | 2.1 | | 1.55 | | 1.04 | | | 2.32 | | | | -0.03 | | | | |  |  |  |  |  |  |  |  |  |  |  |  |  |  |  |  |  |  |  |  |  |  |  |  |  |  |  |  |  |  |  |  |
| P | M84 | Disorders of continuity of bone | 0.9 | 4,454 | | 0.5 | 21,148 | | | 2.16 | 1.49 | | 3.13 | | 1.77 | 1.35 | | 2.32 | | 1.78 | | 1.22 | | | 2.58 | | | | 0.15 | | | | |  |  |  |  |  |  |  |  |  |  |  |  |  |  |  |  |  |  |  |  |  |  |  |  |  |  |  |  |  |  |  |  |
|  | M90 | Osteopathies in diseases classified elsewhere | 0.3 | 4,488 | | 0.2 | 21,253 | | | 2.03 | 1.1 | | 3.74 | | 1.7 | 1.08 | | 2.67 | | 1.7 | | 0.92 | | | 3.14 | | | | -0.17 | | | | |  |  |  |  |  |  |  |  |  |  |  |  |  |  |  |  |  |  |  |  |  |  |  |  |  |  |  |  |  |  |  |  |
|  | **XIV. Diseases of the genitourinary system (N00-N99)** | | | |  | | | | | | | | | | | |  | |  | | | |  | | |  | | | |  | | | | |  | | | |  | | | |  | | | |  | | | |  | | | |  | | | |  | | | |  |  |  |
|  | N81 | Female genital prolapse | 0.1 | 4,507 | | 0 | 21,249 | | | 3.51 | 0.84 | | 14.69 | | 2.83 | 1.16 | | 6.92 | | 2.83 | | 0.68 | | | 11.85 | | | | -0.39 | | | | |  |  |  |  |  |  |  |  |  |  |  |  |  |  |  |  |  |  |  |  |  |  |  |  |  |  |  |  |  |  |  |  |
|  | **XVIII. Symptoms, signs and abnormal clinical and laboratory findings, not elsewhere classified (R00-R99)** | | | | | | | | | | | | |  | | | | | | | | | | | | | | | | | | |  | | | | |  | | | |  | | | |  | | | |  | | | |  | | | |  | | | |  | | | |
|  | R06 | Abnormalities of breathing | 15.7 | 2,911 | | 15.4 | 15,166 | | | 1.18 | 1.07 | | 1.3 | | 1.02 | 0.93 | | 1.11 | | 1.02 | | 0.91 | | | 1.14 | | | | -0.13 | | | | |  |  |  |  |  |  |  |  |  |  |  |  |  |  |  |  |  |  |  |  |  |  |  |  |  |  |  |  |  |  |  |  |
|  | R17 | Unspecified jaundice | 0.3 | 4,483 | | 0.2 | 21,160 | | | 1.91 | 1.04 | | 3.5 | | 1.57 | 1 | | 2.48 | | 1.58 | | 0.86 | | | 2.89 | | | | -0.25 | | | | |  |  |  |  |  |  |  |  |  |  |  |  |  |  |  |  |  |  |  |  |  |  |  |  |  |  |  |  |  |  |  |  |
|  | R18 | Ascites | 1.4 | 4,454 | | 0.9 | 21,095 | | | 1.88 | 1.41 | | 2.51 | | 1.56 | 1.25 | | 1.94 | | 1.57 | | 1.17 | | | 2.10 | | | | 0.11 | | | | |  |  |  |  |  |  |  |  |  |  |  |  |  |  |  |  |  |  |  |  |  |  |  |  |  |  |  |  |  |  |  |  |
|  | R30–R39 | Symptoms and signs involving the urinary system | 6.9 | 3,233 | | 6.6 | 15,279 | | | 1.22 | 1.06 | | 1.41 | | 1.05 | 0.93 | | 1.19 | | 1.05 | | 0.91 | | | 1.22 | | | | -0.15 | | | | |  |  |  |  |  |  |  |  |  |  |  |  |  |  |  |  |  |  |  |  |  |  |  |  |  |  |  |  |  |  |  |  |
|  | R39 | Other symptoms and signs involving the urinary system | 2 | 4,321 | | 1.9 | 20,413 | | | 1.31 | 1.04 | | 1.65 | | 1.09 | 0.9 | | 1.32 | | 1.1 | | 0.87 | | | 1.39 | | | | -0.22 | | | | |  |  |  |  |  |  |  |  |  |  |  |  |  |  |  |  |  |  |  |  |  |  |  |  |  |  |  |  |  |  |  |  |
| P | R40 | Somnolence, stupor, and coma | 0.6 | 4,499 | | 0.5 | 21,246 | | | 1.67 | 1.1 | | 2.52 | | 1.38 | 1 | | 1.91 | | 1.39 | | 0.92 | | | 2.10 | | | | -0.17 | | | | |  |  |  |  |  |  |  |  |  |  |  |  |  |  |  |  |  |  |  |  |  |  |  |  |  |  |  |  |  |  |  |  |
|  | R44 | Other symptoms and signs involving general sensations and perceptions | 0.1 | 4,502 | | 0 | 21,264 | | | 5.89 | 1.88 | | 18.42 | | 4.72 | 2.68 | | 8.32 | | 4.73 | | 1.52 | | | 14.67 | | | | 0.27 | | | | |  |  |  |  |  |  |  |  |  |  |  |  |  |  |  |  |  |  |  |  |  |  |  |  |  |  |  |  |  |  |  |  |
| P | R56 | Convulsions, NEC | 1.9 | 4,353 | | 1.6 | 20,782 | | | 1.46 | 1.14 | | 1.85 | | 1.19 | 0.98 | | 1.45 | | 1.2 | | 0.94 | | | 1.53 | | | | -0.13 | | | | |  |  |  |  |  |  |  |  |  |  |  |  |  |  |  |  |  |  |  |  |  |  |  |  |  |  |  |  |  |  |  |  |
| W | R65 | Systemic inflammatory response Syndrome | 2.5 | 4,389 | | 2.3 | 20,986 | | | 1.31 | 1.07 | | 1.61 | | 1.11 | 0.93 | | 1.31 | | 1.11 | | 0.9 | | | 1.37 | | | | -0.17 | | | | |  |  |  |  |  |  |  |  |  |  |  |  |  |  |  |  |  |  |  |  |  |  |  |  |  |  |  |  |  |  |  |  |
|  | R68 | Other general symptoms and signs | 0.4 | 4,460 | | 0.3 | 21,055 | | | 1.91 | 1.14 | | 3.22 | | 1.57 | 1.07 | | 2.32 | | 1.58 | | 0.94 | | | 2.65 | | | | -0.15 | | | | |  |  |  |  |  |  |  |  |  |  |  |  |  |  |  |  |  |  |  |  |  |  |  |  |  |  |  |  |  |  |  |  |
|  | R95–R99 | Ill-defined and unknown causes of mortality | 0.6 | 4,514 | | 0.3 | 21,297 | | | 2.4 | 1.53 | | 3.77 | | 1.99 | 1.45 | | 2.73 | | 2 | | 1.27 | | | 3.13 | | | | 0.18 | | | | |  |  |  |  |  |  |  |  |  |  |  |  |  |  |  |  |  |  |  |  |  |  |  |  |  |  |  |  |  |  |  |  |
|  | R99 | Other ill-defined and unspecified causes of mortality | 0.5 | 4,514 | | 0.3 | 21,297 | | | 2.48 | 1.53 | | 4.01 | | 2.06 | 1.47 | | 2.88 | | 2.06 | | 1.28 | | | 3.34 | | | | 0.17 | | | | |  |  |  |  |  |  |  |  |  |  |  |  |  |  |  |  |  |  |  |  |  |  |  |  |  |  |  |  |  |  |  |  |
|  | **XIX. Injury, poisoning and certain other consequences of external causes (S00-T98)** | | | | | | | | | | |  | | | | | | | | | | | | | | |  | | | |  | | | | |  | | | |  | | | |  | | | |  | | | |  | | | |  | | | |  | | | |  |  |
|  | S30–S39 | Injuries to the abdomen, lower back, lumbar spine, and pelvis | 4.1 | 3,152 | | 4.1 | 14,804 | | | 1.22 | 1.01 | | 1.48 | | 0.99 | 0.84 | | 1.16 | | 0.99 | | 0.81 | | | 1.20 | | | | -0.28 | | | | |  |  |  |  |  |  |  |  |  |  |  |  |  |  |  |  |  |  |  |  |  |  |  |  |  |  |  |  |  |  |  |  |
| P | S32 | Fracture of the lumbar spine and pelvis | 1.6 | 4,343 | | 1 | 20,523 | | | 1.97 | 1.51 | | 2.58 | | 1.59 | 1.3 | | 1.95 | | 1.6 | | 1.22 | | | 2.10 | | | | 0.16 | | | | |  |  |  |  |  |  |  |  |  |  |  |  |  |  |  |  |  |  |  |  |  |  |  |  |  |  |  |  |  |  |  |  |
|  | S40–S49 | Injuries to the shoulder and upper arm | 1.8 | 3,770 | | 1.5 | 17,985 | | | 1.45 | 1.11 | | 1.89 | | 1.17 | 0.94 | | 1.45 | | 1.17 | | 0.9 | | | 1.53 | | | | -0.18 | | | | |  |  |  |  |  |  |  |  |  |  |  |  |  |  |  |  |  |  |  |  |  |  |  |  |  |  |  |  |  |  |  |  |
| P | S42 | Fracture of the shoulder and upper arm | 0.4 | 4,463 | | 0.2 | 21,124 | | | 2.62 | 1.48 | | 4.65 | | 2.12 | 1.43 | | 3.15 | | 2.12 | | 1.2 | | | 3.76 | | | | 0.1 | | | | |  |  |  |  |  |  |  |  |  |  |  |  |  |  |  |  |  |  |  |  |  |  |  |  |  |  |  |  |  |  |  |  |
|  | S70–S79 | Injuries to the hip and thigh | 1.7 | 4,152 | | 1.2 | 19,599 | | | 1.8 | 1.38 | | 2.35 | | 1.48 | 1.2 | | 1.81 | | 1.48 | | 1.13 | | | 1.94 | | | | 0.08 | | | | |  |  |  |  |  |  |  |  |  |  |  |  |  |  |  |  |  |  |  |  |  |  |  |  |  |  |  |  |  |  |  |  |
|  | S70 | Superficial injury of the hip and thigh | 0.7 | 4,393 | | 0.3 | 20,702 | | | 2.34 | 1.52 | | 3.61 | | 1.92 | 1.41 | | 2.62 | | 1.93 | | 1.25 | | | 2.98 | | | | 0.16 | | | | |  |  |  |  |  |  |  |  |  |  |  |  |  |  |  |  |  |  |  |  |  |  |  |  |  |  |  |  |  |  |  |  |
| P | S72 | Fracture of the femur | 0.7 | 4,460 | | 0.4 | 21,075 | | | 2.21 | 1.45 | | 3.36 | | 1.82 | 1.34 | | 2.47 | | 1.82 | | 1.2 | | | 2.78 | | | | 0.11 | | | | |  |  |  |  |  |  |  |  |  |  |  |  |  |  |  |  |  |  |  |  |  |  |  |  |  |  |  |  |  |  |  |  |
| P | S82 | Fracture of the lower leg, including ankle | 0.3 | 4,425 | | 0.2 | 20,869 | | | 2 | 1.04 | | 3.84 | | 1.57 | 0.96 | | 2.57 | | 1.57 | | 0.82 | | | 3.03 | | | | -0.3 | | | | |  |  |  |  |  |  |  |  |  |  |  |  |  |  |  |  |  |  |  |  |  |  |  |  |  |  |  |  |  |  |  |  |
|  | T25 | Burn and corrosion of the ankle and foot | 0.2 | 4,478 | | 0.1 | 21,174 | | | 2.56 | 1.05 | | 6.22 | | 2.07 | 1.11 | | 3.84 | | 2.07 | | 0.85 | | | 5.04 | | | | -0.25 | | | | |  |  |  |  |  |  |  |  |  |  |  |  |  |  |  |  |  |  |  |  |  |  |  |  |  |  |  |  |  |  |  |  |
|  | **XX. External causes of morbidity and mortality (V01-Y98)** | | | | | | |  | | | | | | | | | | | | |  | | |  | | | |  | | | |  | | | | |  | | | |  | | | |  | | | |  | | | |  | | | |  | | | |  | | | | |
|  | X50–X57 | Overexertion, travel, and privation | 0.1 | 4,515 | | 0 | 21,295 | | | 16.98 | 1.76 | | 163.4 | | 14.15 | 8.03 | | 24.93 | | 14.16 | | 1.47 | | | 136.15 | | | | 0.26 | | | | |  |  |  |  |  |  |  |  |  |  |  |  |  |  |  |  |  |  |  |  |  |  |  |  |  |  |  |  |  |  |  |  |

*W is for Well-known immune-related AEs (dark shade), and P is for Potential ICI-related AEs (light shade).

**The IC value is statistically significant when it is greater than zero.

†Please note that in case of some AE signals in which the incidence of AEs (%) seems higher in the non-ICI group than in the ICI group, the HR can be greater than 1 because the HR reflects the person-year, not only the population.

Abbreviations: KCD: Korean Standard Classification of Diseases, AE: adverse events, HR: hazard ratio, CI: confidence interval, PCR: proportional claims ratio, COR: claims odds ratio, IC: information component lower credible interval, NEC: not elsewhere classified
